# Cardiac adverse events associated with remdesivir in COVID-19 patients: a systematic review and meta-analysis of randomised controlled trials

**DOI:** 10.1136/bmjopen-2024-089977

**Published:** 2025-07-18

**Authors:** Chengliang Yang, Linda Lapp, Alain Amstutz, Matthias Briel, Casey P. Shannon, Hedi Zhao, Estefania Espin, Sara Assadian, Mustafa Toma, Scott J. Tebbutt

**Affiliations:** 1 Prevention of Organ Failure (PROOF) Centre of Excellence,Providence Research, St Paul's Hospital, University of British Columbia, Vancouver, British Columbia, Canada; 2 Centre for Heart Lung Innovation, Providence Research, St Paul's Hospital, University of British Columbia, Vancouver, British Columbia, Canada; 3 Division of Respiratory Medicine, Department of Medicine, University of British Columbia, Vancouver, British Columbia, Canada; 4 CLEAR Methods Center, Division of Clinical Epidemiology, Department of Clinical Research, University of Basel, Basel, Switzerland; 5 Oslo Center for Biostatistics and Epidemiology, Oslo University Hospital, Oslo, Norway; 6 Bristol Medical School, University of Bristol, Bristol, UK; 7 Department of Surgery, The University of British Columbia, Vancouver, British Columbia, Canada; 8 Division of Cardiology, St. Paul's Hospital, University of British Columbia, Vancouver, British Columbia, Canada

**Keywords:** COVID-19, Meta-Analysis, Safety, SARS-CoV-2 Infection, Systematic Review, Adverse events

## Abstract

**Abstract:**

**Objectives:**

To evaluate whether remdesivir is associated with cardiac adverse events (CAEs), addressing concerns raised by basic experiments, clinical case reports and observational studies.

**Design:**

Systematic review and meta-analysis.

**Data sources:**

MEDLINE and Embase, searched from January 2020 to December 2023.

**Study selection:**

Randomised controlled trials (RCTs) comparing remdesivir with placebo or standard care in patients with COVID-19, with a primary focus on cardiac safety.

**Eligibility criteria for selecting studies:**

We included RCTs that evaluated the safety of remdesivir in patients with COVID-19 . Eligible studies were those that compared remdesivir with placebo or standard care in adult patientsCOVID-19 . Inclusion criteria emphasised safety outcomes, particularly CAEs, as primary endpoints.

**Data extraction and synthesis:**

Two reviewers independently extracted data. Reporting followed the Preferred Reporting Items for Systematic Reviews and Meta-Analyses (PRISMA)-Harms guidelines. Risk of bias (RoB) was assessed using the Cochrane Collaboration tool. A random-effects model was used for data synthesis. The Grading of Recommendations Assessment, Development and Evaluation (GRADE) approach was applied to assess the certainty of evidence. The primary outcome was the incidence of any CAEs, defined as a composite of all reported cardiac-related harms. Secondary outcomes included specific CAEs such as arrhythmias, heart failure and myocardial disorders.

**Results:**

We identified 1698 studies, of which seven RCTs met the inclusion criteria, comprising a total of 4566 participants. The RoB was assessed across multiple domains, with four RCTs showing low risk and three showing moderate risk in specific areas. Pooled analysis revealed no significant association between remdesivir use and CAEs (RR=0.84, 95% CI: 0.68 to 1.04, p=0.118). Subgroup analyses showed consistent findings across different patient demographics and comorbidities. GRADE assessment indicated moderate certainty for overall CAEs, low certainty for arrhythmias and heart failure (due to imprecision and study-level bias), and very low certainty for myocardial disorders (due to small sample size and indirectness).

**Conclusions:**

Contrary to preliminary concerns and case reports, our meta-analysis found no evidence of a statistically significant association between remdesivir and CAEs among patients with COVID-19 . These findings provide reassurance to clinicians regarding the safety profile of remdesivir in this patient population, supporting its use as an antiviral therapy in the treatment of COVID-19. Further research is warranted to validate these findings and to clarify whether remdesivir may have a neutral or potentially protective effect on cardiac outcomes.

**PROSPERO registration number:**

CRD42022383647.

**STRENGTHS AND LIMITATIONS OF THIS STUDY**

- This study represents the largest systematic review and meta-analysis evaluating remdesivir-associated cardiac adverse events (CAEs), including only randomised controlled trials (RCTs) to ensure high-quality evidence.
- A rigorous risk of bias (RoB) assessment was conducted using the Cochrane RoB 2 tool, minimising potential methodological biases and ensuring study reliability.
- Meta-regression and subgroup analyses examined sex differences and baseline comorbidities, such as cardiovascular disease and diabetes, to identify potential study-level factors influencing CAEs.
- The study was conducted in unvaccinated populations, allowing us to isolate remdesivir’s potential cardiotoxic effects, independent of COVID-19 vaccination-related myocarditis risks.
- Adherence to Preferred Reporting Items for Systematic Reviews and Meta-Analyses guidelines ensured methodological transparency, reproducibility and compliance with best practices in systematic reviews and meta-analyses.

## Introduction

 Remdesivir (GS-5734) was the first antiviral drug approved by the United States Food and Drug Administration (FDA) to be used as a treatment for COVID-19.^1^ It is a prodrug that exerts its antiviral effects by binding to the viral RNA-dependent RNA polymerase, thereby impeding viral replication through premature termination of RNA transcription.^2^

Guidelines from authoritative bodies such as the Centers for Disease Control and Prevention (CDC), the Infectious Diseases Society of America (IDSA) and the WHO advocate for remdesivir usage in individuals with mild-to-moderate COVID-19, who face elevated risk of severe disease.^3^,^5^ Additionally, WHO guidelines include recommendations for the use of remdesivir in severe cases.^6^ However, the National Institute for Health and Care Excellence (NICE) introduces more specific recommendations. NICE recommends remdesivir as an option for treating COVID-19 in hospitals for adults with a high risk of serious illness, and for babies, children and young people if they meet criteria related to age, weight, pneumonia and the need for supplemental oxygen.^7^

A growing body of case reports and clinical studies, both retrospective and prospective, reports instances of cardiac adverse events (CAEs) associated with remdesivir use.^8^,^13^ Both basic science and clinical studies have raised concerns about potential cardiotoxicity associated with remdesivir, highlighting CAEs, including arrhythmias, sinus bradycardia, T-wave abnormalities, atrial fibrillation, prolonged QT interval and isolated cases of cardiac arrest.^14^,^23^ A preliminary prospective study by Attena et al. (2021) was among the first to report an increased incidence of sinus bradycardia in remdesivir-treated patients, suggesting a potential negative chronotropic effect.^24^ It has been hypothesised that remdesivir’s weak inhibition of human mitochondrial RNA polymerase could lead to mitochondrial damage, oxidative/nitrative stress and that its metabolite’s resemblance to ATP could affect cardiovascular function.^25^,^27^

Although several randomised controlled trials (RCTs) have investigated the effectiveness of remdesivir in COVID-19 treatment,^2 6^ a comprehensive cardiac safety profile remains lacking. Previous systematic reviews have primarily focused on clinical efficacy or all-cause adverse events rather than specifically examining remdesivir’s association with CAEs. Moreover, earlier studies included heterogeneous study designs, such as observational cohorts and case series, limiting their ability to establish a causal relationship between remdesivir and cardiac toxicity. To address this gap, we conducted a systematic review and meta-analysis focused specifically on CAEs associated with remdesivir use in COVID-19 patients.^28^ In line with PRISMA-Harms recommendations, we defined harms-related outcomes as any CAEs reported in the included RCTs, with a specific focus on arrhythmias (eg, atrial fibrillation, bradycardia, ventricular tachycardia and QT prolongation), myocardial disorders (eg, myocarditis and myocardial infarction) and heart failure syndromes. This systematic review evaluates the cardiac safety of remdesivir in patients with COVID-19 using the PICOS framework: participants (P): adults diagnosed with COVID-19; intervention (I): remdesivir treatment; comparison (C): placebo or standard care; outcomes (O): incidence of CAEs and study design (S): RCTs. This review assesses whether remdesivir increases the risk of CAEs and examines subgroup differences based on sex, cardiovascular comorbidities and diabetes. By synthesising RCT data, it provides high-quality evidence to support clinical decision-making regarding remdesivir’s cardiac safety profile.

## Methods

This systematic review was reported according to the Preferred Reporting Items for Systematic Reviews and Meta-Analyses for Harms (PRISMA-Harms) guidelines.^29^ The full PRISMA-Harms checklist is provided in online supplemental file 1. The study protocol was published and registered on PROSPERO (CRD42022383647).

### Eligibility criteria

We included RCTs that enrolled patients diagnosed with COVID-19, in the community or at the hospital, and assessed the safety of remdesivir versus placebo or standard of care (online supplemental table 2). Studies had to report cardiac adverse events (CAEs) as an outcome. We excluded non-RCTs (eg, observational studies, case reports, reviews, etc.), animal studies, conference abstracts, non-English studies, patients without a COVID-19 diagnosis and cases where remdesivir was used to treat conditions other than COVID-19. Studies without reported CAEs were included qualitatively but excluded from meta-analysis. Follow-up durations varied, with most trials reporting adverse events up to 28 days.

### Search strategy

A systematic search was undertaken in MEDLINE and Embase, using Ovid, including literature published from the 1 January 2020 to the 31 December 2023. A customised search strategy was conducted for each database, the details of which can be found in the online supplemental table 3. The search strategy was collaboratively developed with an information specialist at the University of British Columbia in Vancouver, Canada. No language restrictions were applied, but only peer-reviewed, full-text articles in English were included. We did not seek unpublished data from regulatory agencies, drug manufacturers or study authors.

### Screening process

Two independent researchers (CY and LL) screened the titles and abstracts using Covidence.^30^ Full-text screening was undertaken by the same two independent reviewers, with a third researcher (EE) consulted in case of disagreement. Studies meeting predefined PICOS-based eligibility criteria were included. For harms-related outcomes, studies were not excluded based on explicit CAE reporting in the title or abstract. If a study met other eligibility criteria but did not mention CAEs explicitly, then a full-text review was conducted to assess the availability of relevant cardiac safety data. When CAE data were missing or unclear, corresponding authors were contacted for clarification. Data extraction was performed independently by two reviewers (CY and LL) using a structured collection form (online supplemental table 4), with risk of bias (RoB) assessment performed following Cochrane guidelines. The final selection of studies was determined through consensus. All included RCTs obtained individual ethical approval, and the University of British Columbia Ethics Committee confirmed that no additional ethical approval was required for this aggregate data meta-analysis, as only pseudo-anonymised data were used.

### RoB assessment and certainty of evidence assessment

The RoB in the included studies was assessed by two reviewers (CY and LL) independently using the Cochrane Collaboration revised tool of RoB 2.0.^31^ In case of disagreement, a third reviewer (EE) was consulted. To enhance transparency in reporting, we additionally evaluated the certainty of evidence using the Grading of Recommendations, Assessment, Development and Evaluations (GRADE) framework. RoB assessments informed the interpretation of findings and were incorporated into the data synthesis.

### Harms-related outcomes

The primary outcome of interest was whether remdesivir was associated with any CAEs in patients with COVID-19. CAEs were defined according to the Medical Dictionary for Regulatory Activities (MedDRA) terminology^32 33^ and included a range of cardiovascular conditions such as cardiac arrhythmias (eg, atrial fibrillation and bradycardia), myocardial disorders (eg, myocarditis and myocardial infarction), heart failure syndromes and conduction system abnormalities. The secondary outcome included analysis of specific harms-related outcomes and subgroup analyses to explore variations in CAE risks based on baseline characteristics. The effect of remdesivir versus placebo and/or standard care on these specific cardiac events was analysed only if those events were present in more than 5% of patients per treatment group to make a meaningful analysis possible.^34^

### Statistical analysis

Descriptive statistics were used to summarise the pooled patient population, reporting participant-weighted median and IQR for numerical variables, and frequencies and percentages for categorical variables. Weights were based on the number of participants contributed by each study. The primary meta-analysis assessed whether remdesivir was associated with CAEs, following the intention-to-treat principle. A random-effects model was used (R package ‘*metafor’* (version 4.4–0),^35^ applying the inverse variance method with restricted maximum likelihood estimator (τ)^2 36^ and 95% confidence intervals calculated using the Hartung–Knapp–Sidik–Jonkman method).^37^ The treatment effects were reported as risk ratios (RR) and accompanying 95% confidence intervals for each study independently as well as overall. To understand the overall treatment effect on cardiac adverse events, the test statistics of the coefficients (Z-value) and the corresponding p-value were reported. The heterogeneity of the studies was also examined by χ^2^ test, for which the estimated amount of residual heterogeneity (τ^2^), I^2^ statistic and its corresponding p-value were reported. We considered heterogeneity to be significant if the p value was <0.10 or the I^2^ statistic was ≥ 50%^38^ and by visual inspection of the resulting forest plots.^39^

Furthermore, exploratory subgroup analyses were conducted using fixed-effects meta-regression based on study-level variables reported across studies: sex, cardiovascular disease, diabetes, chronic lung disease, chronic liver disease and obesity. For these analyses, stratified fixed-effects meta-regression was undertaken to understand whether, and which, study-level factors could drive the measures of effect. Since different studies reported different sets of baseline comorbidities, the meta-regression was carried out in a univariable manner.^40^ The interaction was not tested due to the lack of reporting of baseline co-morbidities for patients with CAEs and the unavailability of individual participant data for most studies included in the review. Meta-regression analysis was also conducted on studies determined to have a low RoB, as per the results of the RoB assessment. For all analyses, R V. 4.2.2 was used.

### Patient and public involvement

No patient or public involvement.

### Role of the funding source

The funders had no role in the study design, data collection, data analysis, data interpretation or writing of the report, or the decision to submit the manuscript for publication.

## Results

### Trials selected

**Figure 1:**
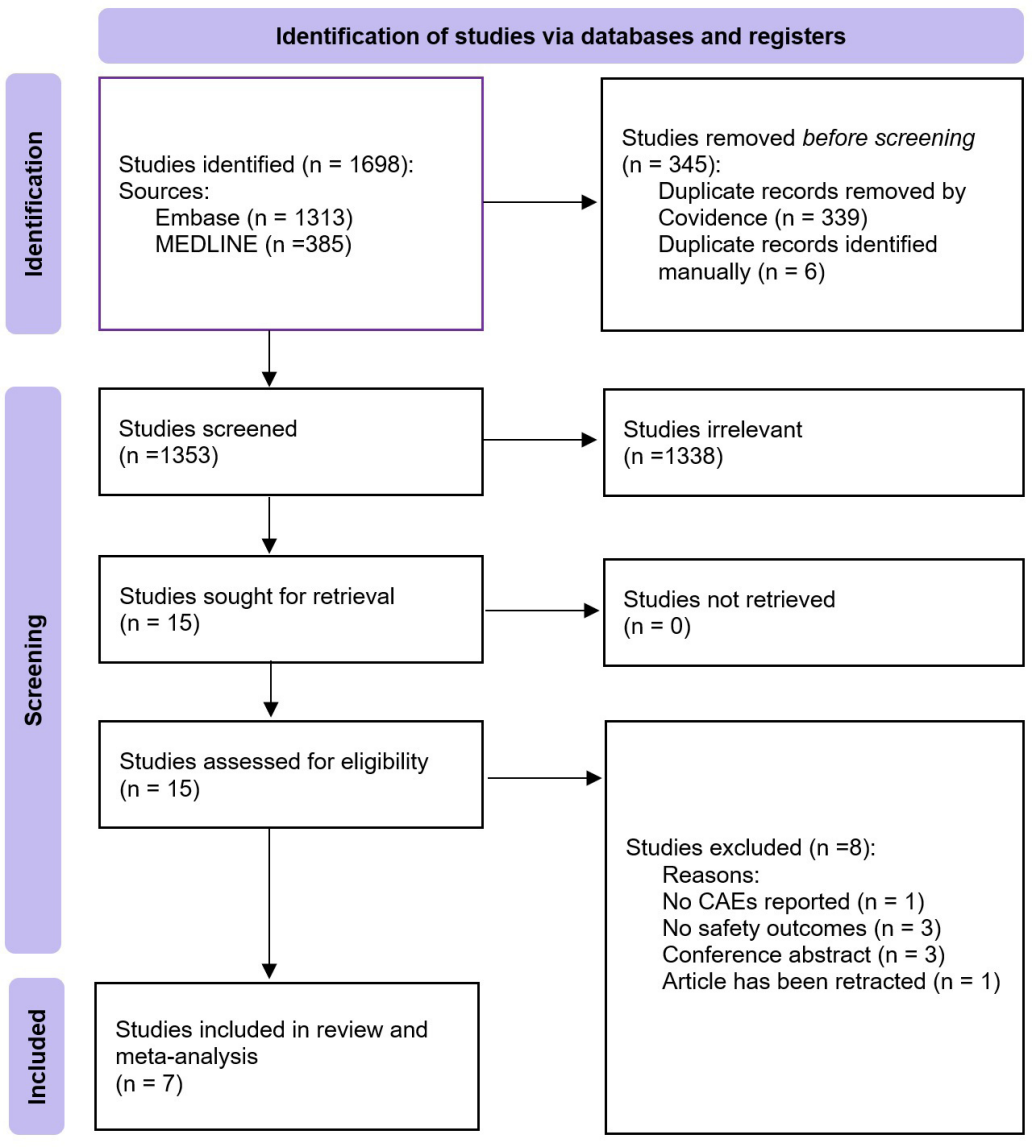
Preferred Reporting Items for Systematic Reviews and Meta-Analyses flow diagram of study selection. Flow diagram illustrating the identification, screening, eligibility assessment, and final inclusion of studies in the systematic review and meta-analysis. CAE, cardiac adverse events.

In total, 1698 studies were identified, 345 of which were duplicated (figure 1). After screening the titles and abstracts of the remaining 1353 studies, 15 studies were deemed to be relevant for full-text assessment.^241^,^54^ Of these, eight studies were excluded due to being conference abstracts (three studies),^47^,^49^ not reporting safety outcomes (three studies),^50 53 54^ not systematically collecting CAEs (one study)^51^ and due to being retracted (one study).^52^ Inclusion and exclusion criteria for the seven included studies in this systematic review and meta-analysis are summarised in online supplemental table 5.^241^,^46^ Further details regarding why the eight studies were excluded from the review can be found in online supplemental table 6.^47^,^54^

As shown in online supplemental table 7, four of the included studies were international (ACTT-1,^41^ Spinner *et al*,^42^ DisCoVeRy^43^ and PINETREE^2^). The number of participants ranged from 99 (NOR-Solidarity^44^ to 1281 CATCO).^45^ Three of the studies were double-blind RCTs (PINETREE,^2^ ACTT-1^41^ and Wang *et al*^46^), and the remainder were open-label RCTs.^42^,^45^ The included studies primarily investigated adult patient populations and six of these included hospitalised patients only.^41^,^46^ One study investigated non-hospitalised patients.^2^ Of note, both the Spinner study and the PINETREE study comprised a very small proportion of young patients, with less than 2% falling within the age range of 12 to 18 years old.^2 42^ All studies were conducted before April 2021, and hence none of the patients had received any COVID-19 vaccine. Three studies included both standard care and placebo as a control,^2 41 46^ and four studies were controlled with standard care only.^42^,^45^ The DisCoVeRy study had the longest follow-up duration of 90 days.^43^

### RoB assessment and certainty of evidence assessment

Among the seven studies evaluated, four were deemed to have low RoB,^4143^,^45^ while three were categorised as having some concerns^2 42 45^ based on the Cochrane RoB 2.0 tool (online supplemental table 8). None were classified as having high RoB. The domain-specific concerns involved the randomisation process or selection of reported results. Pharmaceutical funding was not considered as a formal source of bias, in accordance with Cochrane guidance. Author affiliations and funding disclosures were reported separately in online supplemental table 8 for transparency. To assess the overall certainty of evidence, we applied the Grading of Recommendations, Assessment, Development and Evaluations (GRADE) framework. The certainty of evidence was rated as moderate for overall CAEs, low for arrhythmias and heart failure, and very low for myocardial disorders. These ratings reflected imprecision, small sample sizes and indirectness across studies. The GRADE summary is presented in the online supplemental table 9.

### Patient characteristics

The median age for the total pooled patient population was 58.9 years (IQR : 57.95 to 64.25), with similar ages observed in both remdesivir and control groups (online supplemental table 10). A larger proportion of male patients (62.1%) were included, and around a third had diabetes (33.0%) and 46.4% had hypertension. In terms of co-morbidities, some of the included participants also experienced cardiovascular disease (20.4%), chronic lung disease (13.1%), chronic liver disease (2.3%), obesity (42.4%) and chronic kidney disease (5.5%). Importantly, two studies lacked consistent reporting of baseline co-morbidities such as diabetes and cardiovascular disease among patients experiencing CAEs.^2 42^ Smoking status was not consistently reported by the included studies, and therefore, no analysis could be undertaken regarding this characteristic. Five studies reported baseline COVID-19 severity,^4143^,^46^ with the largest proportion of total number of participants belonging to category 4—hospitalised, requiring supplemental oxygen (37.6%, online supplemental table 10), followed by participants belonging to category 5—hospitalised, receiving non-invasive ventilation or high-flow oxygen devices (23.6%, online supplemental table 10) and participants belonging to category 3—hospitalised, not requiring supplemental oxygen and requiring ongoing medical care (21.5%, online supplemental table 10).

Overall, 306 out of 4656 patients (6.6%) across the seven included RCTs were reported to have experienced CAEs. Among these, 149 (48.7%) were from the remdesivir group, while 157 (51.3%) were from the control group. When looking at the pooled patient characteristics for patients who were reported to have CAEs (online supplemental table 11), the median age overall was 69 years (IQR: 66.0 to 70.0). There does not appear to be a noticeable difference in median ages between remdesivir and control groups (70.0 years vs 69.0 years). In terms of sex, the majority of patients with CAEs tended to be male (78.1%). Regarding co-morbidities, 35.9% of the patients with CAEs had diabetes, 33.3% had cardiovascular disease, 13.9% had chronic lung disease, 2.2% had chronic liver disease, and 42.6% had obesity. Most patients with CAEs were reported to be in the COVID-19 severity grading 3—hospitalised, not requiring supplemental oxygen, and requiring ongoing medical care (COVID-19-related or otherwise) (63.4%). It is worth noting that not all studies provided information on co-morbidities for patients who were reported to have CAEs, and therefore making conclusions on co-morbidities and their effect on CAEs is challenging. Sex was the only patient characteristic that was reported by all studies.

### Primary outcome

**Figure 2:**
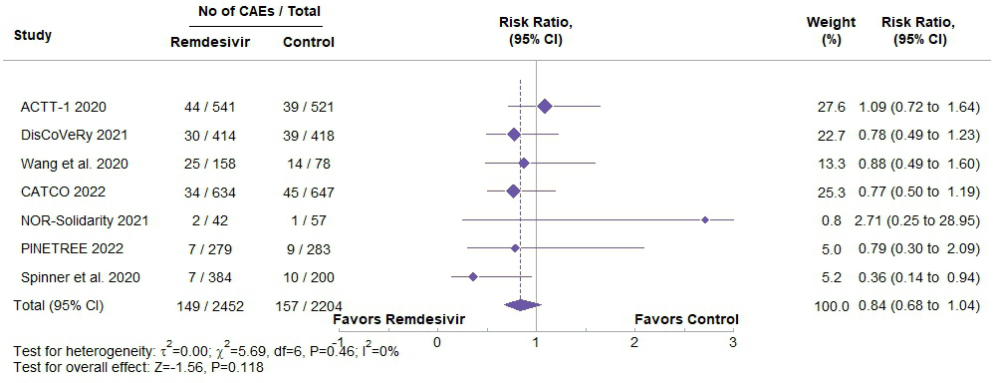
Forest plot of cardiac adverse events (CAEs) in patients treated with remdesivir vs standard care. Pooled risk ratios (RRs) with 95% CIs are shown for each of the seven included randomised controlled trials, along with the total number of events and participants in each group. The diamond represents the overall pooled RR calculated using a random-effects model. No statistically significant difference was observed between groups.

Overall, remdesivir was not associated with a statistically significant increase or decrease in the risk of CAEs compared with control groups (RR=0.84, 95% CI: 0.68 to 1.04), p-value=0.118 (figure 2). The results were similar when assessing only studies deemed as low risk from the RoB assessment (online supplemental figure 1).

**Figure 3:**
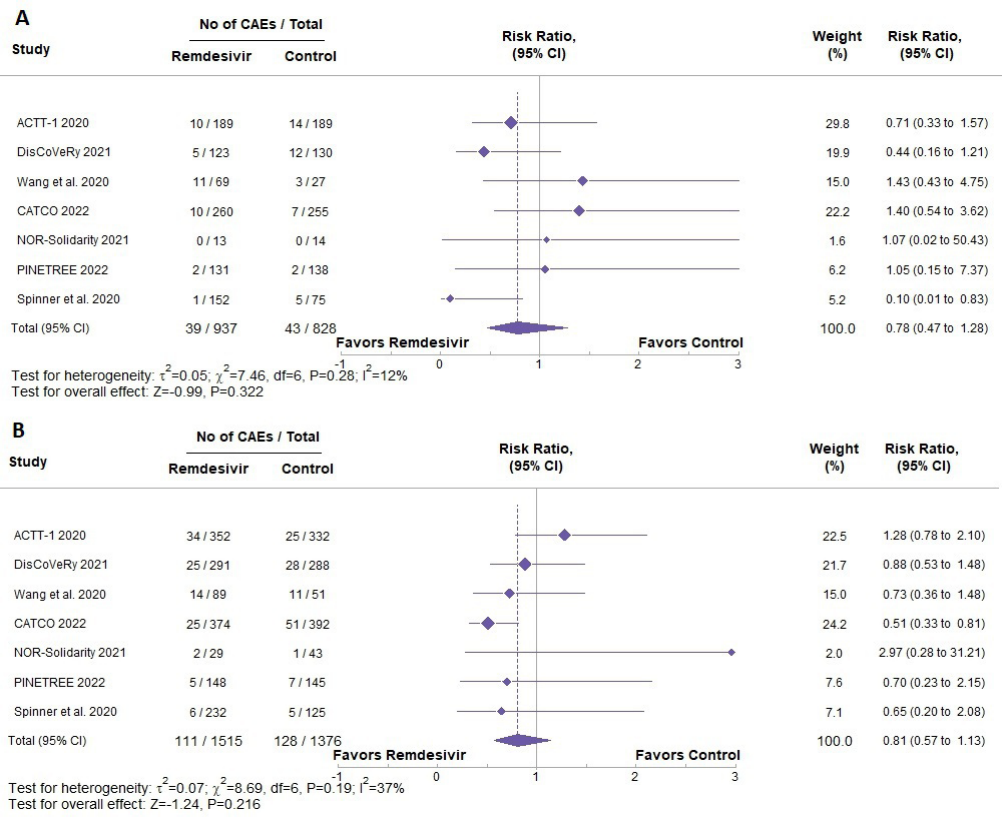
Comparison of female (**A**) and male (**B**) patients having cardiac adverse events based on whether they were treated with remdesivir or standard of care. Risk ratios and 95% confidence intervals are shown for each study. CAE, cardiac adverse events.

When separating patients based on sex (figure 3), the estimated effect of remdesivir on CAEs yielded an RR of 0.78 (95% CI: 0.47 to 1.28; p=0.322) in female patients and 0.81 (95% CI: 0.57 to 1.13; p =0.216) in male patients. The wide confidence intervals encompass both potential benefit and harm, indicating imprecision and lack of conclusive evidence for a sex-specific effect. Only five of the seven studies reported diabetes as a comorbidity. Based on these studies (online supplemental figure 2), patients with diabetes and receiving remdesivir were not at a high risk of CAEs compared with patients with diabetes receiving SOC (RR=0.65, 95% CI: 0.40 to 1.06, p=0.081). Additionally, there was no statistically significant effect of remdesivir on patients with pre-existing cardiovascular disease in terms of having CAEs (online supplemental figure 3) (RR=0.73, 95% CI: 0.38 to 1.41, p=0.351).

### Meta-regression and subgroup analysis

Meta-regression and subgroup analyses (online supplemental table 12) showed no significant effect modification by study RoB, sex or baseline comorbidities. The pooled RR remained non-significant when limited to low-risk-of-bias studies (RR=0.94, 95% CI: 0.71 to 1.23). Subgroup RRs were 0.78 (95% CI: 0.47 to 1.28) for females and 0.81 (95% CI: 0.57 to 1.13) for males. Across comorbidities, including diabetes, cardiovascular disease, chronic lung and liver disease and obesity, no significant interactions were observed, and heterogeneity was generally low.

### Specific cardiac adverse events

The complete list of CAEs reported in studies is shown in online supplemental table 13. All reported events were grouped, using the MedDRA classification system. The most common CAEs were cardiac arrhythmias (including atrial fibrillation, cardiac arrest and tachycardia) with similar proportions across groups, 5.0% in the remdesivir group and 5.6% in the control group. The pooled analysis showed no statistically significant differences between receiving remdesivir or placebo and/or standard care in terms of having a specific CAE (online supplemental table 14).

## Discussion

To the best of our knowledge, this is the first systematic review and meta-analysis specifically investigating the potential cardiotoxicity of remdesivir in adult COVID-19 patients, encompassing both out-of-hospital and in-hospital settings. This systematic review included all accessible evidence from RCTs on CAEs. While several meta-analyses on remdesivir have been published, they predominantly concentrate on the drug’s effectiveness and overall adverse events rather than its potential cardiotoxicity.^55^,^61^

Despite initial concerns arising from basic experiments, clinical case reports and observational studies,^8^,^2325^ our rigorous analysis found no evidence of a statistically significant association between remdesivir treatment and CAEs in COVID-19 patients, although the point estimate (RR=0.84) suggested a possible reduction in risk. Furthermore, subgroup analyses based on sex, cardiovascular disease and diabetes did not indicate any effect modification. A Cochrane review previously found that remdesivir has little to no effect on mortality but may provide modest clinical benefits; however, it did not specifically assess CAEs.^62^ Our study fills this gap by systematically evaluating remdesivir’s cardiovascular safety.

With the presented results in mind, it is worth noting that three studies included in the meta-analysis were flagged for having some concerns for RoB.^2 42 45^ Specifically, the concerns of bias stemmed from the randomisation process in the CATCO study,^45^ and in the selection of reported safety outcomes, in studies conducted by Gilead Sciences (PINETREE study^2^ and Spinner *et al*^42^ (online supplemental table 7)). Although these studies were not at high RoB, the identified domain-specific concerns warrant cautious interpretation of their findings within the broader context of the evidence synthesis. While some studies received funding from pharmaceutical companies, our RoB assessment followed the Cochrane RoB 2.0 framework and did not consider industry involvement as an independent source of bias. Author affiliations and funding disclosures were reported separately in online supplemental table 8 for transparency, in accordance with the Cochrane Handbook.

Evidence suggests that remdesivir has been associated with reduced mortality in hospitalised patients with COVID-19 who require either no or conventional oxygen support.^56^ Despite its widespread use, the potential cardiotoxic effects of remdesivir are still unknown. Therefore, our primary focus in this study was to assess the safety profile of remdesivir in COVID-19 patients, particularly concerning CAEs. Recent research highlights the heightened cardiovascular risks faced by patients with COVID-19 infected with the SARS-CoV-2 virus, encompassing a spectrum of conditions from arrhythmias to heart failure. Studies show the impact of COVID-19 on cardiovascular disease, emphasising arrhythmias, ischaemic and non-ischaemic heart disease, pericarditis, myocarditis, heart failure and thromboembolic disease.^63^,^65^ Autopsy studies confirm SARS-CoV-2’s harmful effect on the heart.^66^ Additionally, pathological studies provided insights into the cardiovascular effects of COVID-19, highlighting myocarditis, interstitial macrophage infiltration and cardiac tropism of SARS-CoV-2.^67 68^ Therefore, it is advisable to regularly monitor signs and symptoms of cardiovascular complications postdiagnosis, extending at least a year post-recovery, especially for infected patients with severe disease.^63^

Accumulating evidence indicates that SARS-CoV-2 infection, affecting various organ systems, not only leads to cardiac injury but also heightens the risk of unfavourable health outcomes in other organs.^69^ These outcomes encompass hospitalisation, conditions impacting the lungs, heart, brain, blood, musculoskeletal system and gastrointestinal system, ultimately contributing to increased mortality.^70 71^ In addition to adverse events like CAEs, following the acute phase of COVID-19, numerous survivors encounter persistent symptoms commonly referred to as ‘long covid’ or ‘post-COVID-19 condition’.^72 73^ Furthermore, a potential correlation may exist between long covid and the prolonged presence of SARS-CoV-2 in individuals experiencing long COVID symptoms.^74 75^ Several studies suggest that remdesivir has the potential to reduce long COVID symptoms.^76^,^78^ However, the findings regarding remdesivir and long covid are non-conclusive.^79^ Consequently, future meta-analysis should specifically assess the association of treatment with remdesivir and the risk of long COVID.

Remdesivir, an intravenous drug administered in a clinical setting, requires more space and staff for infusion compared with other outpatient options. Given the importance of prioritising effective oral antiviral drugs during the ongoing COVID-19 pandemic, VV116 emerges as a potential candidate. VV116, a deuterated remdesivir hydrobromide with oral bioavailability, exhibits favourable safety profiles in phase 1 trials.^80 81^ Phase 3 trials show its non-inferiority to nirmatrelvir–ritonavir in treating COVID-19, especially the B.1.1.529 (omicron) variant.^82^ The promising efficacy and safety of VV116 in managing mild-to-moderate COVID-19, without observed safety concerns, support its therapeutic potential.^83^ Notably, SARS-CoV-2 can infect and replicate in the human body and brain in patients with mild to severe COVID-19 disease.^69 84^ Preclinical studies suggest that VV116 metabolite X1 is poorly distributed in the brain,^80^ a factor not considered in the study.^81 82^ Consequently, well-designed further investigations are necessary for informed decision-making regarding VV116 in patients with COVID-19. The synthesis of these studies contributes to a comprehensive understanding, reinforcing the emphasis on the safety and efficacy of remdesivir, including its deuterated form (VV116), in diverse clinical contexts.

### Strengths and limitations

This study represents the largest systematic review and meta-analysis to date evaluating remdesivir-associated CAEs, incorporating only RCTs to ensure high-quality evidence. A robust RoB assessment was conducted using the Cochrane RoB two tool, ensuring a rigorous evaluation of study quality and minimising potential methodological biases. Furthermore, meta-regression and subgroup analyses were performed to examine sex differences and baseline comorbidities, such as cardiovascular disease and diabetes, identifying potential study-level factors influencing CAEs. Since all included trials were conducted in unvaccinated populations, our findings specifically isolate remdesivir’s potential cardiotoxic effects, independent of COVID-19 vaccination-related myocarditis risks. The study was reported following PRISMA guidelines, ensuring methodological transparency, reproducibility and adherence to best practices in systematic reviews and meta-analyses.

Despite the robustness of this systematic review and meta-analysis, certain limitations apply. First, the RCTs included in this study encompass the early stages of the COVID-19 pandemic, characterised by non-Omicron variants. All participants included in the RCTs study were unvaccinated. The findings may not fully capture the impact of remdesivir on later dominant virus types, such as Omicron variants of SARS-CoV-2,^85 86^ which emerged after the study period. To date, studies have shown a potential risk of heart inflammation (myocarditis and pericarditis) following mRNA vaccination against the SARS-CoV-2. However, the evidence indicates that the benefits of mRNA COVID-19 vaccination outweigh the risk of myocarditis.^87 88^ Our study had a specific focus on the potential cardiotoxicity of remdesivir, and none of the patients included received a COVID-19 vaccine. While this is a potential limitation, it serves to exclude the impact of vaccine side effects from the evaluation, aligning with our primary concern about whether remdesivir has potential cardiotoxic effects. In the RCT study, the settings of the control group and the experimental group are identical, except for the key difference of whether they received remdesivir or placebo/standard treatment. Second, our study exclusively focused on adult COVID-19 patients, leaving the impact of remdesivir on the paediatric population unexplored. Third, all RCTs included in our analysis used intravenous infusion of remdesivir (GS-5734). We did not examine research investigating inhaled remdesivir^89 90^or the oral remdesivir derivative VV116. Fourth, in terms of age, three out of the seven RCTs included in our analysis provided individual information,^40 43 46^ while the remaining four did not, restricting us from exploring the age factor in more detail.^2 42 44 45^ In light of current global vaccination rates, our meta-analysis cannot determine if remdesivir’s impact on CAEs remains constant postvaccination. Furthermore, our analysis of specific CAEs was limited to events occurring in ≥5% of patients per treatment group, a threshold established to ensure statistical reliability and reduce uncertainty associated with rare event analysis. However, we acknowledge that lower-prevalence cardiac events may still be clinically significant and have included this as a limitation in the paper. Fifth, although five studies reported baseline COVID-19 severity using WHO ordinal scales, heterogeneity in reporting and the absence of individual-level severity data precluded formal subgroup meta-analysis by disease severity. As shown in online supplemental tables 10 and 11, 63.4% of patients with CAEs were hospitalised but did not require supplemental oxygen (WHO category 3), suggesting that CAEs were not confined to the most severely ill. Finally, a potential limitation of our study is that our systematic search was conducted through December 2023. Since then, no new RCTs evaluating remdesivir’s cardiac safety have been published. While this suggests that our findings reflect the most up-to-date evidence available, emerging research on other antiviral therapies, such as Nirmatrelvir/ritonavir, may provide further insights into treatment safety profiles. Future studies should provide a more nuanced understanding of remdesivir’s role in diverse patient populations and amidst changing viral landscapes. While our analysis focused on harms-related outcomes (CAEs), differences in event definitions and reporting across trials may have introduced heterogeneity, potentially influencing pooled estimates. Some RCTs reported broad CAE categories, while others provided granular event-level data, impacting comparability. The lack of standardised cardiac safety reporting frameworks underscores the need for future trials to adopt uniform harm reporting methodologies to enhance reliability and reduce bias in safety assessments.

Looking ahead, post-vaccination studies are needed to assess whether remdesivir’s cardiac safety profile remains consistent in immunised populations, particularly as newer variants emerge. In addition, real-world pharmacovigilance data may offer valuable insights into rare or long-term cardiac adverse events that are difficult to capture in RCTs. Future trials should adopt standardised definitions and reporting frameworks for cardiac harms to enhance comparability, improve evidence synthesis and support safer therapeutic decision-making.

### Conclusions

This systematic review and meta-analysis of RCTs provides high-quality evidence that does not support concerns about remdesivir’s cardiotoxic effects. We found no statistically significant association between remdesivir and CAEs, and while the point estimate (RR=0.84) suggests a potential reduction in risk, the CI includes the null, and the results were not statistically significant. GRADE assessment suggests moderate to low certainty for key outcomes. These findings help clarify the cardiac safety profile of remdesivir and support its continued use as an antiviral therapy in COVID-19 treatment. Nonetheless, further research is warranted to assess long-term cardiac outcomes, evaluate safety in vaccinated populations and understand remdesivir’s role across different SARS-CoV-2 variants. Our study offers valuable insights for clinicians, policymakers and regulatory agencies navigating the evolving landscape of COVID-19 therapeutics.

## Supplementary material

10.1136/bmjopen-2024-089977 online supplemental file 1

## Data Availability

Data sharing not applicable as no datasets generated and/or analysed for this study. All data relevant to the study are included in the article or uploaded as supplementary information.
