# Supplementary material for: Cardiac adverse events associated with remdesivir in COVID-19 patients: a systematic review and meta-analysis of randomised controlled trials
Source: BMJ Open. 2025 Jul 18;15(7):e089977. doi: 10.1136/bmjopen-2024-089977 (PMC12273096; doi:10.1136/bmjopen-2024-089977)

**Supplementary Table 1. PRISMA harms checklist**

| Section / topic (page no) | Item | PRISMA checklist item | PRISMA harms (minimum) | | Recommendations for reporting harms in systematic reviews (desirable) | | Check if done |
| --- | --- | --- | --- | --- | --- | --- | --- |
| **Title** | | | | | | | |
| Title (1) | 1 | Identify the report as a systematic review, meta-analysis, or both. | Specifically mention “harms” or other related terms, or the harm of interest in the review. | | — | | Done |
| **Abstract** | | | | | | | |
| Structured summary (2) | 2 | Provide a structured summary including, as applicable: background; objectives; data sources; study eligibility criteria, participants, and interventions; study appraisal and synthesis methods; results; limitations; conclusions and implications of key findings; systematic review registration number. | — | | Abstracts should report any analysis of harms undertaken in the review, if harms are a primary or secondary outcome. | | Done |
| **Introduction** | | | | | | | |
| Rationale (4,5) | 3 | Describe the rationale for the review in the context of what is already known. | — | | It should clearly describe in introduction or in methods section which events are considered harms and provide a clear rationale for the specific harm(s), condition(s), and patient group(s) included in the review. | | Done |
| Objectives (5) | 4 | Provide an explicit statement of questions being addressed with reference to participants, interventions, comparisons, outcomes, and study design (PICOS). | — | | PICOS format should be specified, although in systematic reviews of harms the selection criteria for P, C, and O may be very broad (same intervention may have been used for heterogeneous indications in a diverse range of patients) | | Done |
| **Methods** | | | | | | | |
| Protocol and registration (5) | 5 | Indicate if a review protocol exists, if and where it can be accessed (eg, web address), and, if available, provide registration information including registration number. | — | | No specific additional information is required for systematic reviews of harms. | | Done |
| Eligibility criteria (5) | 6 | Specify study characteristics (eg, PICOS, length of follow-up) and report characteristics (eg, years considered, language, publication status) used as criteria for eligibility, giving rationale. | — | | Report how handled relevant studies (based on population and intervention) when the outcomes of interest were not reported.  Report choices for specific study designs and length of follow-up. | | Done |
| Information sources (6) | 7 | Describe all information sources (eg, databases with dates of coverage, contact with study authors to identify additional studies) in the search and date last searched. | — | | Report if only searched for published data, or also sought data from unpublished sources, from authors, drug manufacturers and regulatory agencies. If includes unpublished data, provide the source and the process of obtaining it. | | Done |
| Search (6) | 8 | Present full electronic search strategy for at least one database, including any limits used, such that it could be repeated. | — | | If additional searches were used specifically to identify adverse events, authors should present the full search process so it can be replicated. | | Done |
| Study selection (6) | 9 | State the process for selecting studies (ie, screening, eligibility, included in systematic review, and, if applicable, included in the meta-analysis). | — | | If only included studies reporting on adverse events of interest, defined if screening was based on adverse event reporting in title/abstract or full text. If no harms reported in the text, report if any attempt was made to retrieve relevant data from authors. | | Done |
| Data collection process (6,7) | 10 | Describe method of data extraction from reports (eg, piloted forms, independently, in duplicate) and any processes for obtaining and confirming data from investigators. | — | | No specific additional information is required for systematic reviews of harms. | | Done |
| Data items (6,7) | 11 | List and define all variables for which data were sought (eg, PICOS, funding sources) and any assumptions and simplifications made. | — | | Report the definition of the harm and seriousness used by each included study (if applicable). Report if multiple events occurred in the same individuals, if this information is available. Consider if the harm may be related to factors associated with participants (eg, age, sex, use of medications) or provider (eg, years of practice, level of training). Specify if information was extracted and how it was used in subsequent results. Specify if extracted details regarding the specific methods used to capture harms (active/passive and timing of adverse event). | | Done |
| Risk of bias in individual studies (6) | 12 | Describe methods used for assessing risk of bias of individual studies (including specification of whether this was done at the study or outcome level), and how this information is to be used in any data synthesis. | — | | The risk of bias assessment should be considered separately for outcomes of benefit and harms. | | Done |
| Summary measures (7) | 13 | State the principal summary measures (eg, risk ratio, difference in means). | — | | No specific additional information is required for systematic reviews of harms. | | Done |
| Synthesis of results (7) | 14 | Describe the methods of handling data and combining results of studies, if done, including measures of consistency (eg, I^2^) for each meta-analysis. | Specify how zero events were handled, if relevant. | |  | | Done |
| Risk of bias across studies (6) | 15 | Specify any assessment of risk of bias that may affect the cumulative evidence (eg, publication bias, selective reporting within studies). | — | | Present the extent of missing information (studies without harms outcomes), any factors that may account for their absence, and whether these reasons may be related to the results. | | Done |
| Additional analyses (7,8) | 16 | Describe methods of additional analyses (eg, sensitivity or subgroup analyses, meta-regression), if done, indicating which were prespecified. | — | | Sensitivity analyses may be affected by different definitions, grading, and attribution of adverse events, as adverse events are typically infrequent or reported using heterogeneous classifications. Report the number of participants and studies included in each subgroup. | | Done |
| **Results** | | | | | | | |
| Study selection (8) | 17 | Give numbers of studies screened, assessed for eligibility, and included in the review, with reasons for exclusions at each stage, ideally with a flow diagram. | — | | If a review addresses both efficacy and harms, display a flow diagram specific for each (efficacy and harm). | | Done |
| Study characteristics (9) | 18 | For each study, present characteristics for which data were extracted (eg, study size, PICOS, follow-up period) and provide the citations. | Define each harm addressed, how it was ascertained (eg, patient report, active search), and over what time period. | | Add additional characteristics to: “P” (population) patient risk factors that were considered as possibly affecting the risk of the harm outcome. “I” (intervention) professional expertise/skills if relevant (for example if the intervention is a procedure). “T” (time) timing of all harms assessments and the length of follow-up. | | Done |
| Risk of bias within studies (9) | 19 | Present data on risk of bias of each study and, if available, any outcome level assessment (see item 12). | — | | Consider the possible sources of biases that could affect the specific harm under consideration within the review. Sample selection, dropouts and measurement of adverse events should be evaluated separately from the outcomes of benefit as described in item 12, above. | | Done |
| Results of individual studies (9,10) | 20 | For all outcomes considered (benefits or harms), present, for each study: (a) simple summary data for each intervention group (b) effect estimates and confidence intervals, ideally with a forest plot. | — | | Report the actual numbers of adverse events in each study, separately for each intervention. | | Done |
| Synthesis of results (10) | 21 | Present results of each meta-analysis done, including confidence intervals and measures of consistency. | Describe any assessment of possible causality. | | If included data from unpublished sources, report clearly the data source and the impact of these studies to the final systematic review. | | Done |
| Risk of bias across studies (9) | 22 | Present results of any assessment of risk of bias across studies (see item 15). | — | | No specific additional information is required for systematic reviews of harms. See item 15 above. | | Done |
| Additional analysis  (10, 11) | 23 | Give results of additional analyses, if done (eg, sensitivity or subgroup analyses, meta-regression (see item 16)). | — | | No specific additional information is required for systematic reviews of harms. | | Done |
| **Discussion** | | | | | | | |
| Summary of evidence (11,12,13) | 24 | Summarise the main findings including the strength of evidence for each main outcome; consider their relevance to key groups (eg, healthcare providers, users, and policy makers). | | — | | No specific additional information is required for systematic reviews of harms. | Done |
| Limitations (13,14,15) | 25 | Discuss limitations at study and outcome level (eg, risk of bias), and at review level (eg, incomplete retrieval of identified research, reporting bias). | | — | | Recognise possible limitations of meta-analysis for rare adverse events (ie, quality and quantity of data), issues noted previously related to collection and reporting. | Done |
| Conclusions (15) | 26 | Provide a general interpretation of the results in the context of other evidence, and implications for future research. | | — | | State conclusions in coherence with the review findings. When adverse events were not identified we caution against the conclusion that the intervention is “safe,” when, in reality, its safety remains unknown. | Done |
| **Funding** | | | | | | | |
| Funding (18) | 27 | Describe sources of funding for the systematic review and other support (eg, supply of data); role of funders for the systematic review. | | — | | No specific additional information is required for systematic reviews of harms. | Done |

| Supplementary Table 2. Inclusion and exclusion criteria of the systematic review and meta-analysis. | | | |
| --- | --- | --- | --- |
| Eligibility Criteria |  | **Included** | **Excluded** |
| Study type |  | Original studies of randomized controlled trials (RCTs) | Any non-RCT (e.g., observational studies, case reports, reviews) |
| Participants |  | All patients diagnosed with COVID-19. Can be either in-hospital or out-patients | Patients who have not been diagnosed with COVID-19. Studies including animals are excluded. |
| Interventions |  | Remdesivir is used to treat COVID-19 compared to placebo.  Remdesivir combined with other drugs is allowed. | Remdesivir is used to treat other diagnoses than COVID-19. |
| Control |  | Placebo or standard care | Non-placebo or no control group. |
| Outcomes |  | Patients with any/at least one cardiac event | If no safety result of the drug is reported. |
| Other |  | Studies in English. | Studies not in English. |

| Supplementary Table 3. Search strategy. | | |
| --- | --- | --- |
| Database | **Search terms** | **Results** |
| EMBASE via OVID | Embase was searched, using the following search terms:  Remdesivir.mp  Remdesivir/  Veklury.mp  GS-5734.mp  COVID-19.mp  Exp coronavirus disease 2019.  SARS-CoV-2.mp  Exp Severe acute respiratory syndrome coronavirus 2/  Coronavirus.mp  Randomi*.mp  Exp randomized controlled trial/  Or/1-4  Or/5-9  Or/10-11  12 and 13 and 14  Search limits: 1 January 2020 to 31 December 2023 | 1313 |
| Medline via OVID | MEDLINE was searched, using the following search terms:  Remdesivir.mp  Veklury.mp  GS-5734.mp  Or/1-3  COVID-19/  SARS-CoV-2/  SARS-CoV-2.mp  Severe Acute Respiratory Syndrome/  Exp Coronavirus/  Coronavirus.mp  Or/5-10  Randomi*.mp  Randomized Controlled Trials as Topic/  Randomized Controlled Trial.pt  Or/12-14  4 and 11 and 5  Search limits: 1 January 2020 to 31 December 2023 | 385 |

| Supplementary Table 4. Structured data collection form. | | | | | | | |
| --- | --- | --- | --- | --- | --- | --- | --- |
| Item | **Study 1** | **Study 2** | **Study 3** | **Study 4** | **Study 5** | **Study 6** | **Study 7** |
| First author |  |  |  |  |  |  |  |
| Year of publication |  |  |  |  |  |  |  |
| Country of study |  |  |  |  |  |  |  |
| Study start |  |  |  |  |  |  |  |
| Study end |  |  |  |  |  |  |  |
| Study design |  |  |  |  |  |  |  |
| Setting |  |  |  |  |  |  |  |
| Type of patient |  |  |  |  |  |  |  |
| The total number of participants |  |  |  |  |  |  |  |
| Number of participants receiving treatment |  |  |  |  |  |  |  |
| Number of participants in the control group |  |  |  |  |  |  |  |
| The dose of treatment |  |  |  |  |  |  |  |
| Participants’ sex |  |  |  |  |  |  |  |
| Participants’age |  |  |  |  |  |  |  |
| Baseline comorbidities |  |  |  |  |  |  |  |
| Smoking status |  |  |  |  |  |  |  |
| Name of cardiac adverse event |  |  |  |  |  |  |  |

| Supplementary Table 5. Detailed inclusion and exclusion criteria for included trials. | | |
| --- | --- | --- |
| Study Author | Inclusion Criteria | Exclusion Criteria |
| ACTT-1  2020 | 1.Admitted to a hospital with symptoms suggestive of COVID-19 infection.  2. Subject (or legally authorized representative) provides informed consent prior to initiation of any study procedures.  3. Subject (or legally authorized representative) understands and agrees to comply with planned study procedures.  4. Male or non-pregnant female adult > / = 18 years of age at time of enrollment.  5. Has laboratory-confirmed SARS-CoV-2 infection as determined by polymerase chain reaction (PCR) or other commercial or public health assay in any specimen, as documented by either or the following:  1). PCR positive in sample collected < 72 hours prior to randomization; OR  Exclusion Criteria:  2). PCR positive in sample collected >/= 72 hours prior to randomization, documented inability to obtain a repeat sample (e.g. due to lack of testing supplies, limited testing capacity, results taking >24 hours, etc.) AND progressive disease suggestive of ongoing SARS-CoV-2 infection.  6. Illness of any duration, and at least one of the following:  1)Radiographic infiltrates by imaging (chest x-ray, CT scan, etc.), OR  2). SpO2 < / = 94% on room air, OR  3). Requiring supplemental oxygen, OR  4). Requiring mechanical ventilation.  7. Women of childbearing potential must agree to either abstinence or use at least one primary form of contraception not including hormonal contraception from the time of screening through Day 29.  8. Agrees to not participate in another clinical trial for the treatment of COVID-19 or SARS-CoV-2 through Day 29. | 1. Alanine Transaminase (ALT) or Aspartate Transaminase (AST) > 5 times the upper limit of normal.  2. Estimated glomerular filtration rate (eGFR) < 30 ml/min (including patients receiving hemodialysis or hemofiltration).  3. Pregnancy or breast feeding.  4. Anticipated discharge from the hospital or transfer to another hospital which is not a study site within 72 hours.  5. Allergy to any study medication. |
| CATCO  2022 | Each participant must meet all of the following inclusion criteria to participate in this study:  1.≥ 18 years of age  2. Has laboratory-confirmed SARS-CoV-2 infection as determined by PCR, or other commercial or public health assay in any specimen, within 14 days prior to randomization.  3. Hospitalized at a participating centre  4. Primary reason for hospitalization or subsequent in-hospital illness is because of acute COVID-19 infection  5. First admission for acute COVID19  In addition, there will be the below intervention-specific inclusion:  Randomization WHO will have no other specific inclusion criteria.  Randomization LSALT will have no other specific inclusion criteria.  Randomization Dex will have the following specific inclusion criteria  1). On 10 days of steroid course and  2). Receiving any supplemental oxygen for 10 days | All participants meeting any of the following exclusion criteria at baseline will be excluded from participation in this study:  1. Anticipated transfer to another hospital, within 72 hours, which is not a study site  2. Expected to not survive beyond 24 hours  3. Receiving one of the study drugs at time of enrolment  In addition, there will be the below intervention-specific exclusions:  Randomization WHO:  These will be drug-specific exclusions; patients will still be eligible for randomization in Randomization WHO to the other available study drugs (in randomization WHO or subsequent randomizations).  Artesunate:  1. Known hypersensitivity to artesunate  Imatinib:  1. Pregnant or breastfeeding;  2. Known hypersensitivity to imatinib;  3.Liver transaminases (either ALT or AST) > 5x upper limit of normal  Infliximab:  1.Known moderate or severe heart failure, per treating clinician, defined as New York Heart Association (NYHA) class III or IV  2.Known or suspected active tuberculosis  3.Known hypersensitivity to infliximab  Randomization LSALT:  1.Known hypersensitivity or prior use of LSALT peptide.  2.Pregnant or breastfeeding  Randomization Dex:  1. Receiving glucocorticoids for a specific, non-COVID-19 indication |
| DisCoVeRy  2022 | 1. Adult ≥18 years of age at the time of enrolment  2. Hospitalized patients with any of the following criteria:  1) The presence of pulmonary rales/crackles on clinical exam OR  2) SpO2 ≤ 94% on room air OR  3) Requirement of supplementary oxygen including high flow oxygen devices or non-invasive ventilation  3. A time between onset of symptoms and randomization of less than 11 days  4. A positive SARS-CoV-2 PCR performed on a NP swab within the 5 days preceding randomization  5. The result of a rapid antigen test performed on a NP swab within the 6 hours preceding randomization  6. Contraceptive use by men or women.  1). Male participants: Contraception for male participants is required; to avoid the transfer of any fluids, all male participants must use a condom from Day 1 and agree to continue for 90 days following administration of IMP.  2). Female participants: Women of child-bearing potential must agree to use contraception for 365 days following administration of IMP | 1. Refusal to participate expressed by patient or legally authorized representative  2. Need for invasive mechanical ventilation and/or ECMO at the time of enrolment  3. Spontaneous blood ALT/AST levels > 5 times the upper limit of normal  4. Glomerular filtration rate (GFR) < 15 mL/min or requiring maintenance dialysis  5. Pregnancy or breast-feeding  6. Anticipated transfer to another hospital, which is not a study site within 72 hours following randomization  7. Known history of allergy or reaction to any component of the study drug formulation.  8. Previous hypersensitivity, infusion-related reaction, or severe adverse reaction following administration of monoclonal or polyclonal antibodies.  9. Any prior receipt of investigational or licensed other mAb/biologic indicated for the prevention of SARS-CoV-2 infection or COVID-19, and for those not vaccinated, expected receipt of vaccine in the 30 days following hospital discharge, according to current recommendation in each country.  10. Any medical condition which, in the judgment of the investigator, could interfere with the interpretation of the trial results or that preludes to protocol adherence. |
| NOR-Solidarity  2021 | 1. Adult patients, 18 years and above  2. Confirmed SARS-2-CoV-2 infection by PCR  3. Admitted to the hospital ward or the ICU  4. Subjects (or legally authorized representative) provides written informed consent prior to initiation of the study | 1. Severe co-morbidity with life expectancy <3 months according to investigators assessment  2. (Aspartate Transaminase/ Alanine Aminotransferase) ASAT/ALAT > 5 times the upper limit of normal  3. Acute co-morbidity within 7 days before inclusion such as myocardial infarction  4. Known intolerance to the available study drugs  5. Pregnancy, possible pregnancy or breast feeding  6. Any reason why, in the opinion of the investigators, the patient should not participate  7. Subject participates in a potentially confounding drug or device trial during the course of the study  8. Prolonged QT interval (>450 ms) |
| PINETREE  2021 | 1. Willing and able to provide written informed consent, (individuals ≥ 18 years of age) or assent (individuals ≥ 12 and < 18 years of age) prior to performing study procedures. Individuals age ≥ 18 years may be enrolled with the consent of a legal representative where permitted according to local law and approved nationally and by the relevant institutional review board (IRB) or independent ethics committee (IEC). For individuals ≥ 12 and < 18 years of age, a parent or legal guardian must be willing and able to provide written informed consent prior to performing study procedures  2. Either:  1). Age ≥ 18 years (at all sites) or aged ≥ 12 and < 18 years of age weighing ≥ 40 kg (where permitted according to local law and approved nationally and by the relevant IRB or IEC with at least 1 pre-existing risk factor for progression to hospitalization (chronic lung disease, hypertension, cardiovascular or cerebrovascular disease, diabetes, obesity (body mass index ≥ 30), immunocompromised, chronic mild or moderate kidney disease, chronic liver disease, current cancer, or sickle cell disease)  2). Or aged ≥ 60 years  3. Severe acute respiratory syndrome (SARS)-coronavirus (CoV)-2 infection confirmed by molecular diagnosis (nucleic acid (polymerase chain reaction (PCR) or antigen testing) ≤ 4 days prior to screening  4. Presence of ≥ 1 symptom(s) consistent with COVID-19 for ≤ 7 days prior to randomization  5. Not currently requiring hospitalization (hospitalization defined as ≥ 24 hours of acute care) | 1. Participation in any other clinical trial of an experimental treatment and prevention for COVID-19  2. Prior hospitalization for COVID-19  3. Treatment with other agents with actual or possible direct antiviral activity against SARS-CoV-2 or administration of any SARS-CoV-2 (or COVID-19) vaccine  4. Requiring oxygen supplementation |
| Spinner  2020 | 1. Willing and able to provide written informed consent prior to performing study procedures (participants ≥ 18 years of age) or assent (participants ≥ 12 and < 18 years of age) prior to performing study procedures. For participants ≥ 12 and < 18 years of age, a parent or legal guardian willing and able to provide written informed consent prior to performing study procedures  2. SARS-CoV-2 infection confirmed by polymerase chain reaction (PCR) test ≤ 4 days before randomization  3. Currently hospitalized and requiring medical care for COVID-19  4. Peripheral capillary oxygen saturation (SpO2) > 94% on room air at screening  5. Radiographic evidence of pulmonary infiltrates | 1. Participation in any other clinical trial of an experimental treatment for COVID-19  2. Concurrent treatment or planned concurrent treatment with other agents with actual or possible direct acting antiviral activity against SARS-CoV-2  3. Requiring mechanical ventilation at screening  4. Alanine Aminotransferase (ALT) or aspartate aminotransferase (AST) > 5 X upper limit of normal (ULN)  5. Creatinine clearance < 50 mL/min using the Cockcroft-Gault formula for participants ≥ 18 years of age {Cockcroft 1976} and Schwartz Formula for participants < 18 years of age |
| Wang  2020 | 1. Age ≥18 years at time of signing Informed Consent Form  2. Laboratory (RT-PCR) confirmed COVID-19.  3. Lung involvement confirmed with chest imaging  4. Hospitalized with a SaO2/SPO2≤94% on room air or Pa02/Fi02 ratio <300mgHg  5. ≤12 days since illness onset  6. Willingness of study participant to accept randomization to any assigned treatment arm.  7. Must agree not to enroll in another study of an investigational agent prior to completion of Day 28 of study. | 1. Physician makes a decision that trial involvement is not in patients' best interest, or any condition that does not allow the protocol to be followed safely.  2. Severe liver disease (e.g. Child Pugh score ≥ C, AST>5 times upper limit)  3. Pregnant or breastfeeding, or positive pregnancy test in a predose examination  4. Patients with known severe renal impairment (estimated glomerular filtration rate ≤30 mL/min/1.73 m2) or receiving continuous renal replacement therapy, hemodialysis, peritoneal dialysis  5. Will be transferred to another hospital which is not the study site within 72 hours.  6. Receipt of any experimental treatment for COVID-19 within the 30 days prior to the time of the screening evaluation. |

| Supplementary Table 6. Characteristics of randomized controlled trials that could not be included in the meta-analysis. | | | | | | | | | |
| --- | --- | --- | --- | --- | --- | --- | --- | --- | --- |
| Study | **No of participant** | **Type of randomized controlled trial** | **Population** | **Recruitment**  **period** | **Intervention** | **Control** | **Primary Endpoint** | **Follow-up**  **duration** | **Reason not included in the meta-analysis** |
| Mahajan et al | 82 | Open-label, single- center, randomized, controlled trial | Adult patients with moderate to severe COVID-19 and not  Mechanically ventilated | June 2020-December 2020 | Intravenous remdesivir  (200 mg day 1, 100 mg day 2-5) | Standard care only | Patient's clinical status at day 5 | 24 days | No adverse events reported, no further reply from authors after initial contact |
| WHO Solidarity | 8275 | Open-label, multicenter, randomized controlled trial | Adults hospitalized with COVID-19 | March 2020-  January 2021 | Intravenous remdesivir  (200 mg day 1, 100 mg day 2-10) | Standard care only | In-hospital mortality | ≥ 60 days | They did not systematically collect CAEs, confirmed with the author. |
| Abd-Elsalam et al | 200 | Open-label, multicenter, randomized, controlled trial | Adults hospitalized with laboratory-confirmed COVID-19 | June 2020-December 2020 | Intravenous remdesivir  (200 mg day 1, 100 mg day 2-5 or 100 mg day 2-10) | Standard care only | Length of hospital stay | 16 days | This article has been retracted by the Journal based on concerns raised by a third party. |
| Criner et al | 584 | Open-label, randomized, controlled trial | Adults hospitalized with laboratory-confirmed  COVID-19 | Unclear | Intravenous remdesivir  (200 mg day 1, 100 mg day 2-10) | Standard care only | Adverse events and laboratory abnormalities | 28 days | Conference abstract |
| Hill et al | 562 | Double-blind, , randomized, placebo controlled trial | Adult patients with moderate to severe COVID-19 | Unclear | Intravenous remdesivir  (200 mg day 1, 100 mg day 2-3) | Standard care and placebo | Proportion of participants with treatment-emergent adverse events | 28 days | Conference abstract |
| Marty et al | 584 | Open-label, randomized, controlled trial | Adults hospitalized with laboratory-confirmed COVID-19 | Unclear | Intravenous remdesivir  (200 mg day 1, 100 mg day 2-3) | Standard care only | Distribution of clinical status assessed on the 7-point ordinal scale on study day 11 | 28 days | Conference abstract |
| Hormati,et al | 141 | One-blind, randomized, placebo-controlled trial | Critically sick adult and child COVID-19 patients and with laboratory-confirmed COVID-19 | May 2020- September 2020 | For adults: intravenous remdesivir  (200 mg day 1, 100 mg day 2-5)  For *pediatric patients:* intravenous remdesivir  (5 mg/kg day 1, 5.2 mg/kg mg day 2-5) | Standard care and placebo | The efficacy of  remdesivir with supportive care alone in the treatment of critically  sick adult and child COVID-19 patients | Unclear | No adverse events reported, no further reply from authors after initial contact |
| Jittamala et al | 131 | Open-label, randomized, controlled adaptive platform trial | Low-risk adults with early symptomatic CIVID-19 | September 2021- June 2022 | Intravenous remdesivir  (200 mg day 1, 100 mg day 2-5) | Standard care only | The rate of viral clearance on day 7 | 7 days | No cardiac adverse event reported |

| Table 7. Characteristics of included trials. | | | | | | | | | |
| --- | --- | --- | --- | --- | --- | --- | --- | --- | --- |
| Study | No of participant | Type of RCT | Population | Female sex -no. (%) | Median  age (IQR),  years | Intervention | Control | Primary Endpoint | Follow-up  duration |
| ACTT-1  (2020) | 1062 | Double-blind, multicenter, randomized, placebo-controlled with stratification based on disease severity. | Adults hospitalized with laboratory-confirmed  Covid-19 | 378 (35.6) | 59 (49-70) | Intravenous remdesivir  (200 mg day 1, 100 mg day 2-10) | Standard care and placebo | The time to recovery | 28 days |
| CATCO  (2022) | 1281 | Open-label, multicenter, randomized controlled trial | Adults hospitalized with laboratory-confirmed  Covid-19 | 515 (40.2) | 66 (54-77) | Intravenous remdesivir  (200 mg day 1, 100 mg day 2-10) | Standard care only | In-hospital mortality | 60 days |
| DisCoVeRy (2022) | 843 | Open-label, adaptive, multicenter, randomized, controlled trial | Adults hospitalized with laboratory-confirmed COVID-19 and requiring oxygen or ventilator support. | 268 (30.4) | 64 (54–73) | Intravenous remdesivir  (200 mg day 1, 100 mg day 2-10) | Standard care only | The patient's clinical status at day 15, was assessed using the seven-point ordinal scale from the WHO Master Protocol. | 90 days |
| NOR-Solidarity (2021) | 99 | Open-label, adaptive, multicenter, randomized, controlled trial | Adults hospitalized with laboratory-confirmed  Covid-19 | 27 (27.3) | 58 (48–72) | Intravenous remdesivir  (200 mg day 1, 100 mg day 2-10) | Standard care only | All-cause hospital mortality | 60 days |
| PINETREE (2021) | 562 | Double-blind, multicenter, randomized, placebo-controlled trial | Non-hospitalized  patients with COVID-19 who had symptom onset within the previous  7 days and who had at least one risk factor for disease progression | 269  (47.9) | 50 (35-65) | Intravenous remdesivir  (200 mg day 1, 100 mg day 2-3) | Standard care and placebo | Hospitalization related to COVID-19 or death from any cause by day 28. | 28 days |
| Spinner et al (2020) | 584 | Open-label, multicenter, randomized, controlled trial | Adults hospitalized with laboratory-confirmed COVID-19 | 227 (38.9) | 57 (45–66) | Intravenous remdesivir  (200 mg day 1, 100 mg day 2-3) | Standard care only | Distribution of clinical status assessed on the 7-point ordinal scale on study day 11 | 28 days |
| Wang et al  (2020) | 236 | Double-blind, multicenter, randomized, placebo-controlled trial | Adult patients admitted to hospital with severe COVID-19* | 96 (40.7) | 65 (56-71) | Intravenous remdesivir  (200 mg day 1, 100 mg day 2-3) | Standard care and placebo | Time to clinic improvement within 28 days after randomization, characterized by the six-point scale | 28 days |

| Supplementary Table 8. Risk of bias assessment for cardiac adverse events using the Cochrane RoB 2.0 tool, with additional disclosure of author affiliations for transparency. | | | | | | |
| --- | --- | --- | --- | --- | --- | --- |
| Name  (year) | **Randomization**  **process** | **Assignment to**  **intervention** | **Missing outcome**  **data** | **Measurement of**  **outcome** | **Selection of**  **reported results** | **Author affiliations** |
| ACTT-1  (2020) | Low risk | Low risk | Low risk | Low risk | Low risk | One of the authors has affiliation with Gilead Sciences. |
| CATCO  (2022) | Some concerns* | Low risk | Low risk | Low risk | Low risk | Some authors report receiving grants and fees from different pharmaceutical companies, such as AstraZeneca, Nplex Biosciences, GEn1E, Gilead, Merck etc outside of the published work. |
| DisCoVeRy  (2022) | Low risk | Low risk | Low risk | Low risk | Low risk | Study authors report receiving funds and grants from pharmaceutical companies, such as Gilead Sciences, Merck,AstraZeneca, etc, outside of the published study. |
| NOR-Solidarity (2021) | Low risk | Low risk | Low risk | Low risk | Low risk | NA |
| PINETREE  (2021) | Low risk | Low risk | Low risk | Low risk | Some concerns** | One author is affiliated with Gilead Sciences, which might bring some bias to the reporting of results |
| Spinner et al  (2020) | Low risk | Low risk | Low risk | Low risk | Some concerns** | The study was sponsored by Gilead Sciences and all authors are affiliated with Gilead Sciences |
| Wang et al  (2020) | Low risk | Low risk | Low risk | Low risk | Low risk | The study thanks Gilead Sciences for providing the study drugs. |

Note: Only the five standard domains of the Cochrane RoB 2.0 tool were used to assess risk of bias. Author affiliations and funding disclosures are presented for transparency but were not included in the formal RoB 2.0 assessment, in accordance with the Cochrane Handbook (Chapter 8).

| Supplementary Table 9. GRADE Summary of findings | | | | | | |
| --- | --- | --- | --- | --- | --- | --- |
| Outcome | **No. of RCTs** | \| **No. of participants** \| \| --- \| | \| **Effect Estimate (RR, 95% CI)** \| \| --- \| | \| **Certainty of evidence** \| \| --- \| | **Comment** | **Interpretation** |
| \| **Cardiac adverse events (CAEs)** \| \| --- \| | 7 | \| 4,566 \| \| --- \| | 0.84 (0.68 to 1.04) | Moderate | Imprecision, study-level bias | The moderate-certainty evidence suggests that remdesivir is unlikely to increase overall cardiac adverse events in COVID-19 patients |
| \| **Arrhythmias** \| \| --- \| | 5 | 3,210 | 0.78 (0.47 to 1.28) | Low | Inconsistency, imprecision | The low-certainty evidence for arrhythmias indicates that further research is required to clarify these risks. |
| \| **Heart failure** \| \| --- \| | 4 | 2,789 | 0.81 (0.57 to 1.13) | Low | Small sample size, inconsistencies | The low-certainty evidence for heart failure indicates that further research is required to clarify these risks. |
| \| **Myocardial disorders** \| \| --- \| | 3 | 1,895 | 0.73 (0.38 to 1.41) | Very low | Small sample size, indirectness, heterogeneity | The very low-certainty evidence for myocardial disorders reflects the small sample size and indirect reporting, necessitating further high-quality trials. |

Low: low-certainty evidence; Moderate: moderate-certainty evidence; Very low: very low-certainty evidence; RCTs: randomized controlled trial

Some included RCTs had methodological limitations, such as incomplete blinding or industry sponsorship, potentially influencing effect estimates. For the imprecision, wide confidence intervals suggest uncertainty in effect estimates. For the inconsistency, variability in definitions and reporting of specific CAEs across RCTs. For the indirectness: some RCTs reported cardiac events as secondary outcomes rather than predefined endpoints. A limited number of RCTs reported detailed cardiac safety outcomes, potentially affecting the overall certainty of evidence.

| Supplementary Table 10. Baseline characteristics of pooled patients from seven randomized controlled trials. | | | | | |
| --- | --- | --- | --- | --- | --- |
| Characteristic | **Total** | **Remdesivir** | **Control** | **Studies Reporting (%)** | **N_Total_, N_Remdesivir_, N_Control_** |
| Age, years  (median, IQR) | 58.9  (57.95 - 64.25) | 59.7  (57.8 - 64.0) | 59.2  (57.55 - 64.00) | 7 (100%) | 4656, 2452, 2204 |
| Sex |  |  |  | 7 (100%) | 4656, 2452, 2204 |
| Male | 2891 (62.09%) | 1515 (52.40%) | 1376 (47.60%) |  |  |
| Female | 1765 (37.91%) | 937 (53.09%) | 828 (46.91%) |  |  |
| Diabetes | 1534 (32.95%) | 801 (52.22%) | 733 (47.78%) | 7 (100%) | 4656, 2452, 2204 |
| Hypertension | 1179 (46.36%) | 662 (47.15%) | 517 (45.39%) | 5 (71.4%) | 2543, 1404, 1139 |
| Cardiovascular Disease | 951 (20.43%) | 525 (55.21%) | 426 (44.79%) | 7 (100%) | 4656, 2452, 2204 |
| Chronic Lung Disease | 579 (13.10%) | 299 (13.03%) | 280 (13.17%) | 6 (85.7%) | 4420, 2294, 2126 |
| Chronic Liver Disease | 85 (2.27%) | 38 (2.03%) | 47 (2.51%) | 4 (57.1%) | 3737, 1868, 1869 |
| Obesity | 1083 (42.39%) | 545 (42.71%) | 538 (42.06%) | 4 (57.1%) | 2555, 1276, 1279 |
| Chronic Kidney Disease | 136 (5.54%) | 64 (5.19%) | 72 (5.89%) | 3 (42.9%) | 2456, 1234, 1222 |
| COVID-19 Severity |  |  |  | 5 (71.4%) | 3995, 2131, 1864 |
| 1 - Ambulatory, no activity limitation | 0 (0.00%) | 0 (0.00%) | 0 (0.00%) |  |  |
| 2 - Ambulatory, activity limitation | 125 (3.13%) | 71 (3.33%) | 54 (2.90%) |  |  |
| 3 - Hospitalized, not requiring supplemental oxygen, requiring ongoing medical care (Covid-19-related or otherwise) | 859 (21.50%) | 420 (19.71%) | 439 (23.55%) |  |  |
| 4 - Hospitalized, requiring supplemental oxygen | 1504 (37.65%) | 803 (37.68%) | 701 (37.61%) |  |  |
| 5 - Hospitalized, receiving non-invasive ventilation or high-flow oxygen devices | 941 (23.55%) | 558 (26.18%) | 383 (20.55%) |  |  |
| 6 - Hospitalized, receiving invasive mechanical ventilation or ECMO | 554 (13.87%) | 270 (12.67%) | 284 (15.24%) |  |  |

| Supplementary Table 11. Pooled patient characteristics among those reported with cardiac adverse events. | | | | | |
| --- | --- | --- | --- | --- | --- |
| Characteristic | **Total** | **Remdesivir** | **Control** | **Studies Reporting (%)** | **N_Total_, N_Remdesivir_, N_Control_** |
| Age, years (median, IQR) | 69.0  (66.0 - 70.0) | 70.0  (66.0 - 70.0) | 69.0  (68.5 - 70.5) | 5 (71.4%) | 273, 135, 138 |
| Sex |  |  |  | 7 (100.0%) | 306, 149, 157 |
| Male | 239 (78.10%) | 111 (74.50%) | 128 (81.53%) |  |  |
| Female | 82 (26.80%) | 39 (26.17%) | 43 (27.39%) |  |  |
| Diabetes | 98 (35.90%) | 39 (28.89%) | 59 (42.75%) | 5 (71.4%) | 273, 135, 138 |
| Cardiovascular Disease | 78 (33.33%) | 31 (28.18%) | 47 (37.90%) | 4 (57.1%) | 234, 110, 124 |
| Chronic Lung Disease | 38 (13.92%) | 15 (11.11%) | 23 (16.67%) | 5 (71.4%) | 273, 135, 138 |
| Chronic Liver Disease | 6 (2.20%) | 4 (2.96%) | 2 (1.45%) | 5 (71.4%) | 273, 135, 138 |
| Obesity | 66 (42.58%) | 36 (47.37%) | 30 (37.97%) | 3 (42.9%) | 155, 76, 79 |
| COVID-19 Severity |  |  |  | 2 (28.6%) | 82, 36, 46 |
| 1 - Ambulatory, no activity limitation | 0 (0.00%) | 0 (0.00%) | 0 (0.00%) |  |  |
| 2 - Ambulatory, activity limitation | 6 (7.32%) | 2 (5.56%) | 4 (8.70%) |  |  |
| 3 - Hospitalized, not requiring supplemental oxygen, requiring ongoing medical care (Covid-19-related or otherwise) | 52 (63.41%) | 19 (52.78%) | 33 (71.74%) |  |  |
| 4 - Hospitalized, requiring supplemental oxygen | 24 (29.27%) | 6 (16.67%) | 18 (39.13%) |  |  |
| 5 - Hospitalized, receiving noninvasive ventilation or high-flow oxygen devices | 14 (17.07%) | 10 (27.78%) | 4 (8.70%) |  |  |
| 6 - Hospitalized, receiving invasive mechanical ventilation or ECMO | 0 (0.00%) | 0 (0.00%) | 0 (0.00%) |  |  |

| Supplementary Table 12. Summary of risk ratios, meta-regression estimates, and heterogeneity testing for cardiac adverse events by subgroup | | | | | | | | |  |
| --- | --- | --- | --- | --- | --- | --- | --- | --- | --- |
| Model | **Number of studies included** | **RR (95% CI)** | **Meta-regression results** | | **Heterogeneity testing** | | | | |
|  |  |  | Estimate | Standard Error | P-value | tau^2 | I^2 | P-value | |
| Predicting CAEs overall, all studies included | 7 | 0.84 (0.68 - 1.04) | -0.1733 | 0.11 | 0.1180 | <0.001 | <0.001% | 0.4583 | |
| Predicting CAEs overall, low bias studies included | 4 | 0.94 (0.71 - 1.23) | -0.0666 | 0.14 | 0.6300 | <0.001 | <0.001% | 0.5813 | |
| When including specific comorbidities |  |  |  |  |  |  |  |  | |
| Cardiovascular Disease | 4 | 0.73 (0.38 - 1.41) | -0.3090 | 0.33 | 0.3510 | 0.2011 | 49.39% | 0.1541 | |
| Sex: Female | 7 | 0.78 (0.47 - 1.28) | -0.2510 | 0.25 | 0.3221 | 0.0547 | 11.91% | 0.2800 | |
| Sex: Male | 7 | 0.81 (0.57 - 1.13) | -0.2152 | 0.17 | 0.2156 | 0.0705 | 36.51% | 0.1915 | |
| Diabetes | 5 | 0.65 (0.40 - 1.06) | -0.4362 | 0.25 | 0.0812 | 0.0831 | 27.03% | 0.3459 | |
| Chronic Lung Disease | 4 | 0.60 (0.30 - 1.21) | -0.5096 | 0.36 | 0.1560 | <0.001 | <0.001% | 0.6771 | |
| Obesity | 3 | 1.18 (0.74 - 1.86) | 0.1618 | 0.23 | 0.4898 | <0.001 | <0.001% | 0.9220 | |
| Chronic Liver Disease | 3 | 1.28 (0.25 - 6.55) | 0.2443 | 0.83 | 0.7698 | <0.001 | <0.001% | 0.5004 | |

| Supplementary Table 13. Cardiac adverse events reported in included trials investigating remdesivir for COVID-19 patients. | | | |
| --- | --- | --- | --- |
| Study Author | Reported Cardiac Adverse Events | Remdesivir Group  no. | Control Group  no. |
| ACTT-1  2020 | Acute myocardial infarction  Acute coronary syndrome  Arrhythmia  Atrial fibrillation  Atrioventricular block  Bradycardia  Cardiac arrest  Cardiac failure  Cardiac tamponade  Cardiogenic shock  Cardiomyopathy  Defect conduction intraventricular  Myocardial infarction  Myocarditis  Myocardial ischaemia  Palpitations  Pulseless electrical activity  Sinus node dysfunction  Supraventricular tachycardia  Tachyarrhythmia  Ventricular fibrillation  Ventricular tachycardia | 1  1  5  13  2  2  11  1  0  0  0  1  4  0  1  1  1  1  3  1  1  4 | 1  0  1  11  0  1  11  1  1  3  1  0  0  1  0  0  0  0  5  1  0  1 |
| CATCO  2022 | Arrhythmia  Atrial Fibrillation  Bradycardia  Cardiac arrest  Cardiac ischemia  Heart failure  Right Bundle Branch Block | 1  10  5  16  7  1  1 | 6  3  2  14  12  0  1 |
| DisCoVeRy  2022 | Arrhythmia  Atrial fibrillation  Atrial flutter  Auriculoventricular block  Bradycardia  Cardiac arrest  Cardiac failure  Fibrillation auriculaire  Hypercardiac output  Heart rhythm disorder  Right cardiogenic shock  Sinus tachycardia  Tachycardia  Stress cardiomyopathy  Supra ventricular tachycardia  Ventricular tachycardia | 4  7  2  1  4  1  1  0  0  1  0  1  6  1  1  0 | 5  11  0  0  5  3  3  1  1  0  1  1  7  0  0  1 |
| NOR-Solidarity  2021 | Arrhythmia  Ventricular tachycardia | 1  1 | 0  1 |
| PINETREE  2021 | Palpitations  Cardiac failure congestive  Angina pectoris  Acute myocardial infarction  Acute left ventricular failure  Mitral valve prolapse  Tachycardia  Bradycardia  Atrial Fibrillation | 2  1  1  0  1  0  1  0  2 | 3  0  1  1  0  1  2  2  0 |
| Spinner  2020 | Atrioventricular block complete  Cardiac failure  Myocardial infarction  Left ventricular dysfunction  Myocarditis  Tachycardia  Bradycardia  Atrial fibrillation  Supraventricular tachycardia  Cardiac arrest  Ventricular extrasystoles | 1  1  0  0  1  1  0  2  0  0  1 | 1  0  1  1  0  3  1  2  1  2  0 |
| Wang  2020 | Arrhythmia  Atrial fibrillation  Atrial Flutter  Cardiac arrest  Heart failure  Ischemic cardiomyopathy  left anterior fascicular block  Myocardial infarction  Myocardial ischemia  Palpitation  Premature Ventricular Contraction  Prolonged QT interval  Sinus arrhythmia  Sinus bradycardia  Tachyarrhythmia  Tachycardia | 2  1  2  1  9  0  1  1  1  2  1  1  1  1  0  1 | 0  2  1  0  6  1  0  0  0  0  0  3  0  0  1  0 |

| Supplementary table 14. Risk ratios (RR) and their respective 95% confidence intervals (CI) and P-values for each specific cardiac adverse event group. | | | | |
| --- | --- | --- | --- | --- |
| Cardiac adverse events | **Remdesivir**  **n (%)** | **Control**  **n (%)** | **RR (95% CI)** | **P-value** |
| Cardiac Arrhythmias | 122 (4.98%) | 123 (5.58%) | 0.86 (0.62-1.18) | 0.3389 |
| Heart Failures | 15 (0.61%) | 17 (0.77%) | 0.60 (0.30-1.23) | 0.1646 |
| Myocardial Disorders | 4 (0.16%) | 12 (0.54%) | 0.37 (0.13-1.07) | 0.0676 |
| Coronary Artery Disorders | 13 (0.53%) | 21 (0.95%) | 0.59 (0.30-1.17) | 0.1316 |
| Cardiac Disorders, Signs or Symptoms | 5 (0.20%) | 3 (0.14%) | 1.08 (0.35-3.34) | 0.8957 |
| Mitral Valvular Disorders | 1 (0.04%) | 0 (0.00%) | 1.08 (0.26-4.53) | 0.9108 |

Supplementary Figure 1. Forest plot showing the risk ratios of a patient having a cardiac adverse event based on whether they were treated with remdesivir or standard of care/placebo for each study deemed as a low risk study from the Risk of Bias assessment.


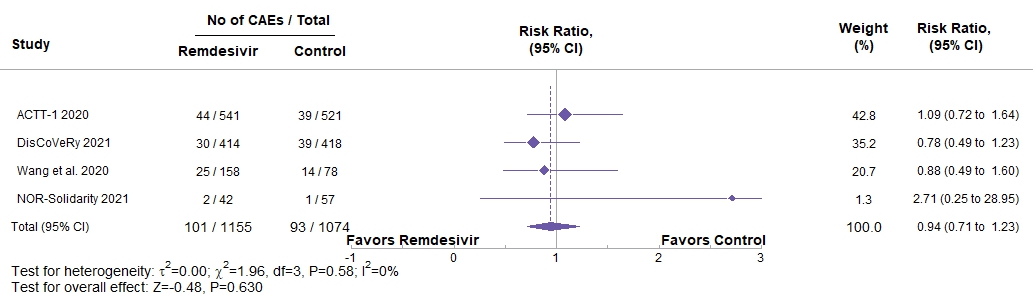


Supplementary Figure 2. Comparison of patients with diabetes having cardiac adverse events based on whether they were treated with remdesivir or standard care/placebo.


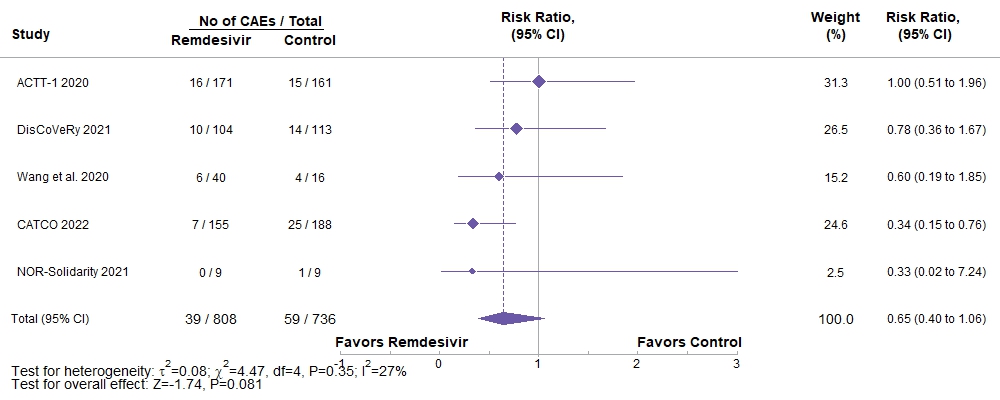


Supplementary Figure 3. Comparison of patients with cardiovascular disease having cardiac adverse events based on whether they were treated with remdesivir or standard care/placebo.


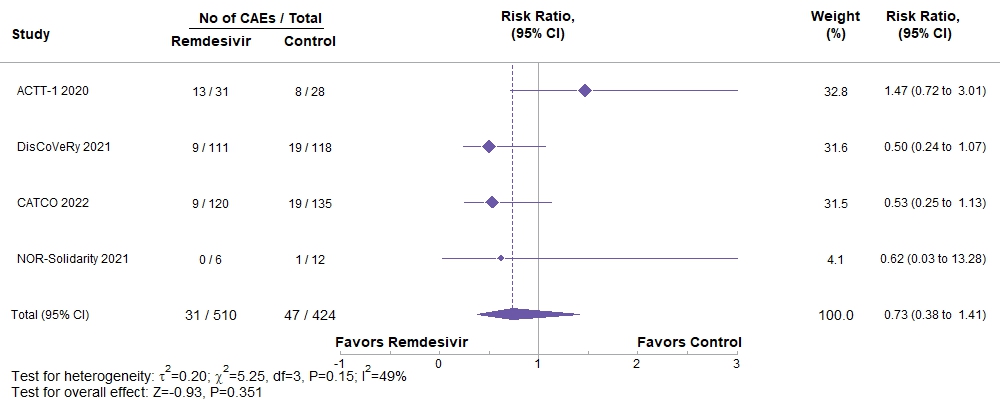

Supplement: online supplemental file 1 [file bmjopen-15-7-s001.docx]
