# Supplementary material for: Cardiac adverse events associated with remdesivir in COVID-19 patients: a systematic review and meta-analysis of randomised controlled trials
Source: BMJ Open. 2025 Jul 18;15(7):e089977. doi: 10.1136/bmjopen-2024-089977 (PMC12273096; doi:10.1136/bmjopen-2024-089977)
Supplement: Supplementary file 2 [file bmjopen-2024-089977.reviewer_comments.pdf]

# PEER REVIEW HISTORY

BMJ Open publishes all reviews undertaken for accepted manuscripts. Reviewers are asked to complete a checklist review form and are provided with free text boxes to elaborate on their assessment. These free text comments are reproduced below.

## ARTICLE DETAILS

### Title (Provisional)

Cardiac adverse events associated with remdesivir in COVID-19 patients: a systematic review and meta-analysis of randomized controlled trials

### Authors

Yang, Chengliang; Lapp, Linda; Amstutz, Alain; Briel, Matthias; Shannon, Casey P.; Zhao, Hedi; Espin, Estefania; Assadian, Sara; Toma, Mustafa; Tebbutt, Scott

---

## VERSION 1 - REVIEW

---

| | |
|---|---|
| **Reviewer** | **1** |
| **Name** | **Chootrakool, Hathaikan** |
| **Affiliation** | **Mahidol University, Biostatistics** |
| **Date** | **01-Jul-2024** |
| **COI** | **None** |

---

Why do you review only two databases?

---

| | |
|---|---|
| **Reviewer** | **2** |
| **Name** | **Albani, Stefano** |
| **Affiliation** | **Ospedale Regionale Umberto Parini** |
| **Date** | **14-Jul-2024** |
| **COI** | **none** |

---

The authors performed a meta-analysis of randomized trial on the association between Remdesivir and adverse cardiac events. The authors found no relationship between the medication and acquired cardiac disorders.

I commend the authors for the subsequent aspects:

1) The objectives of the current meta-analysis are clearly stated and a clinically relevant and focused study question is provided.

2) A comprehensive literature search was conducted, reasonable limitations were placed on search.

3) The characteristics of the studies listed are correctly reported and inclusion and exclusion criteria are clearly provided for the considered studies. The number of excluded studies and reasons for exclusion is clearly stated.

4) The risk for publication bias was assessed using the Cochrane Collaboration tool.

5) Whole sections of the manuscript are appropriately written as contents and reasonable conclusions are provided and appropriately commented in the discussion section.

An important preliminary study conducted by Attena and colleagues https://doi.org/10.1161/CIRCEP.121.009811 should be appropriately cited in the introduction as this work was a trailblazer for further studies in this field, especially at the beginning of covid19 pandemic.

---

| | |
|---|---|
| **Reviewer** | **3** |
| **Name** | **Díaz-Alvarez, Agustín** |
| **Affiliation** | **University Hospital of Salamanca** |
| **Date** | **21-Aug-2024** |
| **COI** | **No competing interests** |

---

Sticking exclusively to the statistical analysis, I think the authors have done an excellent job. They have correctly followed the steps for the elaboration of a Meta-analysis:

- Well-defined working hypothesis.

- Selection of the study population: they have described the sources of information, the search criteria, as well as the inclusion criteria.

- In the statistical analysis they have selected the random effects method, and have correctly established the significance values, as well as the graphical representation of the risk ratios.

- The analysis of biases using the Cochrane Collaboration is interesting and accurate.

In summary, this is a meta-analysis that has been correctly carried out from a methodological point of view.

---

| | |
|---|---|
| **Reviewer** | **4** |
| **Name** | **Rahouma, Mohamed** |
| **Affiliation** | **Weill Cornell Medicine, Cardiothoracic Surgery** |
| **Date** | **07-Oct-2024** |

| | |
|---|---|
| **COI** | **No. Not applicable** |

Yang et al reported their work named “Association between remdesivir and cardiac adverse events

among patients with COVID-19: a systematic review and meta-analysis of randomized controlled trials” and concluded” Contrary to preliminary concerns and case reports, our meta-analysis found no statistically significant association between remdesivir and cardiac adverse events among COVID-19 patients. These findings provide reassurance to clinicians regarding the safety profile of remdesivir in this patient population, supporting its continued use in clinical practice. Further research is warranted to validate these findings and explore potential mechanisms underlying remdesivir’s cardiotoxic effects.”. I have the following comments:

- Please spell out any abbreviations at their first time eg CAE in the abstract, CDC, IDSA, WHO.

- Line 170: Please specify that this is the “weighted” median.

- Language revision is essential.

- Please add results of meta-regression to the supplements in a table format.

- Please add strength and limitations at the end of discussion section.

- Table 2 and 3: Please transfer them to the supplements. Pleaseadd “Outcomes summary” table including outcomes, number of studies, number of patients, effect estimate (RR (%%CI),P value), and heterogeneity (I^2 and P value). Please report both random and fixed effect model results in that table. I think just edit the table in page 56 and move it to the main manuscript and add a title for that.

- Supplementary Table 7: Please delete the column “Overall risk of bias”. You can delete the related text in the abstract as well.

| | |
|---|---|
| **Reviewer** | **5** |
| **Name** | **Martin, James** |
| **Affiliation** | **University of Birmingham, Institute of Applied Health Research** |
| **Date** | **14-Jan-2025** |
| **COI** | **None** |

Thank you for submitting this systematic review to BMJ Open. I have a number of comments, which are listed below.

You include some acronyms in the abstract, such as CAE and PRISMA, without first introducing what they stand for.

You have a lot of text in the abstract under "eligibility criteria for selecting studies" which doesn't really fit under this heading. You may want to include an additional heading and move some of the text, so that it reads better.

In the introduction, it is mentioned about case reports and clinical studies having reported a relationship between cardiac adverse events and remdesivir use. If there has been a number of trials published previously investigating the effectiveness of remdesivir, can you add more context on what this review brings that previous literature hasn't already summarised.

On lines 124 – 127, you provide a definition of a systematic review and meta-analysis. This is not needed here.

Can you remove the words "conducted and" on lines 130 as the PRISMA guidelines are for reporting, not describing how to conduct a systematic review.

You included RCTs that enrolled patients diagnosed with COVID-19. Was the method for diagnosing COVID part of an eligibility criteria?

You have searched MEDLINE and Embase to identify studies. I wonder whether you have looked at other reviews of remdesivir and COVID-19 to check that you have identified all appropriate studies.

In the outcomes of interest, In the outcomes of interest, you indicate that a cardiac adverse event was defined as per the Medical Dictionary for Regulatory Activities terminology, but then do not give what this definition in.

What is the difference between your primary is the difference between your primary outcome of interest "cardiac adverse event" and your second outcome of interest "specific reported cardiac adverse event"?

Why were the effects of remdesivir vs placebo only looked at if the events occurred in more than 5% of patients per group? It could still be a meaningful analysis with a prevalence less than this.

For the subgroup analyses, can you list all baseline co-morbidities that were considered in the meta-regression.

---

## VERSION 1 - AUTHOR RESPONSE

**Reviewer #1 (Dr. Hathaikan Chootrakool, Mahidol University)**

**Comment 1.**Why do you review only two databases?

Response: We used MEDLINE and Embase because they are the most comprehensive sources for clinical trials, ensuring broad coverage of relevant randomized controlled trials (RCTs) (PMID: 30860595). While other databases exist, these two databases provide the most extensive indexing of biomedical literature, including peer-reviewed journal articles relevant to our research question.

**Reviewer #2 (Dr. Stefano Albani, Ospedale Regionale Umberto Parini)**

**Comment 1:**The objectives of the current meta-analysis are clearly stated and a clinically relevant and focused study question is provided.

Response: Thank you for your positive feedback. We appreciate your acknowledgment of the clarity and clinical relevance of our study question.

**Comment 2:** A comprehensive literature search was conducted, reasonable limitations were placed on search.

Response: We appreciate this recognition. Our search strategy was designed to be exhaustive while maintaining a focus on high-quality, peer-reviewed randomized controlled trials (RCTs). The search was conducted in two major medical databases (MEDLINE and Embase), with clear inclusion and exclusion criteria outlined in the Methods section and Supplementary Tables 2 and 3.

**Comment 3:** The characteristics of the studies listed are correctly reported and inclusion and exclusion criteria are clearly provided for the considered studies. The number of excluded studies and reasons for exclusion is clearly stated.

Response: Thank you for this positive feedback.

**Comment 4:** The risk for publication bias was assessed using the Cochrane Collaboration tool.

Response: Yes, we assessed the risk of bias using the Cochrane Collaboration's RoB 2 tool, as described in the Methods section. The results of this assessment are presented in Supplementary Table 8.

**Comment 5:**Whole sections of the manuscript are appropriately written as contents and reasonable conclusions are provided and appropriately commented in the discussion section.

Response: We sincerely appreciate your positive assessment of our manuscript's structure, discussion, and conclusions.

**Comment 6:** An important preliminary study conducted by Attena and colleagues https://doi.org/10.1161/CIRCEP.121.009811 should be appropriately cited in the

introduction as this work was a trailblazer for further studies in this field, especially at the beginning of covid19 pandemic.

Response: Thank you for this valuable suggestion. We have reviewed the study by Attena et al. (2021) and now cite it in the Introduction. Their prospective study was among the first to report an increased incidence of sinus bradycardia in remdesivir-treated patients, suggesting a potential negative chronotropic effect, possibly linked to remdesivir's ATP-like structure. This study contributed to early discussions on remdesivir's cardiac effects, highlighting the need for further systematic evaluation, as addressed in our review.

**Reviewer# 3 (Dr. Agustín Díaz - Alvarez, University Hospital of Salamanca)**

**Comment 1:** Sticking exclusively to the statistical analysis, I think the authors have done an excellent job. They have correctly followed the steps for the elaboration of a Meta- analysis:

- Well-defined working hypothesis.
- Selection of the study population: they have described the sources of information, the search criteria, as well as the inclusion criteria.
- In the statistical analysis they have selected the random effects method, and have correctly established the significance values, as well as the graphical representation of the risk ratios.
- The analysis of biases using the Cochrane Collaboration is interesting and accurate.

In summary, this is a meta-analysis that has been correctly carried out from a methodological point of view.

**Response**: Thank you for your positive feedback. We appreciate your recognition of our rigorous methodology, statistical approach, and bias assessment.

**Reviewer# 4 (Dr. Mohamed Rahouma, Weill Cornell Medicine, National Cancer Institute)**

**Comment 1:** Please spell out any abbreviations at their first time eg CAE in the abstract, CDC, IDSA, WHO.

**Response:** We have revised the manuscript to ensure that all abbreviations are fully spelled out at their first mention in the abstract and main text.

**Comment 2:** Line 170: Please specify that this is the "weighted" median.

**Response:** We have clarified this in the manuscript by specifying that the reported value is the "weighted median."

**Comment 3:** Language revision is essential.

**Response:** Thank you for your comments. We have carefully revised the manuscript for clarity, grammar, and conciseness to improve readability.

**Comment 4:** Please add results of meta-regression to the supplements in a table format.

**Response:** We have added a supplementary table 12 summarizing the meta-regression results.

**Comment 5:** Please add strength and limitations at the end of discussion section.

Response: We have added a "Strengths and Limitations" section at the end of the Discussion, summarizing key methodological aspects.

**Comment 6:** Table 2 and 3: Please transfer them to the supplements. Please add "Outcomes summary" table including outcomes, number of studies, number of patients, effect estimate (RR (%%CI),P value), and heterogeneity (I^2 and P value). Please report both random and fixed effect model results in that table. I think just edit the table in page 56 and move it to the main manuscript and add a title for that.

**Response** : As suggested, Tables 2 and 3 have been moved to the supplementary materials. In their place, we have created a new outcomes summary table (now Supplementary Table 12), which includes outcomes, number of studies and patients, effect estimates (RR with 95% CI and p-value), heterogeneity ($I^2$ and p-value), and results from both random-effects and fixed-effects models

**Comment 7:** Supplementary Table 7: Please delete the column "Overall risk of bias". You can delete the related text in the abstract as well.

**Response:** We have removed the "Overall risk of bias" column from Supplementary Table 8 and deleted the corresponding text from the abstract.

**Reviewer# 5 (Dr. James Martin, University of Birmingham)**

**Comment 1:** You include some acronyms in the abstract, such as CAE and PRISMA,

without first introducing what they stand for.

**Response:** We have revised the abstract to ensure that all acronyms, including CAE (cardiac adverse events) and PRISMA (Preferred Reporting Items for Systematic Reviews and Meta-Analyses), are fully spelled out at first mention.

**Comment 2:** You have a lot of text in the abstract under "eligibility criteria for selecting studies" which doesn't really fit under this heading. You may want to include an additional heading and move some of the text, so that it reads better.

**Response:** We have revised the abstract structure by adjusting the placement of information under appropriate subheadings to improve clarity and readability.

**Comment 3:** In the introduction, it is mentioned about case reports and clinical studies having reported a relationship between cardiac adverse events and remdesivir use. If there has been a number of trials published previously investigating the effectiveness of remdesivir, can you add more context on what this review brings that previous literature hasn't already summarised.

**Response:** We have expanded the Introduction to clarify how this review builds upon previous research. While earlier studies have reported case series and observational data on remdesivir-associated CAEs, our systematic review and meta-analysis is the first to synthesize evidence exclusively from RCTs, providing a higher level of evidence regarding remdesivir's potential cardiotoxic effects. Additionally, we emphasize that previous studies have lacked a comprehensive cardiac safety profile for remdesivir in COVID-19 patients.

**Comment 4:** On lines 124 – 127, you provide a definition of a systematic review and meta-analysis. This is not needed here.

**Response:** Thank you for the suggestion. We have removed it as recommended.

**Comment 5:** Can you remove the words "conducted and" on lines 130 as the PRISMA guidelines are for reporting, not describing how to conduct a systematic review.

**Response:** We have corrected this statement to accurately reflect that PRISMA guidelines pertain to reporting, not the conduct of a systematic review.

**Comment 6:** You included RCTs that enrolled patients diagnosed with COVID-19. Was

the method for diagnosing COVID part of an eligibility criteria?

Response: Yes, the method for diagnosing COVID-19 was explicitly included as an eligibility criterion across all included RCTs. Each study required laboratory-confirmed SARS-CoV-2 infection, primarily through RT-PCR or equivalent molecular testing, before patient enrollment. This ensured that all participants had a confirmed COVID-19 diagnosis before receiving remdesivir or control treatments. For detailed study-specific diagnostic methods, please refer to Supplementary Table 5.

**Comment 7:** You have searched MEDLINE and Embase to identify studies. I wonder whether you have looked at other reviews of remdesivir and COVID-19 to check that you have identified all appropriate studies.

**Response:** We appreciate the reviewer's question. In addition to conducting a systematic search in MEDLINE and Embase, we cross-referenced our study selection with existing systematic reviews and meta-analyses on remdesivir and COVID-19 to confirm the inclusion of all relevant RCTs. This additional step ensured that no eligible trials were overlooked. Our search strategy was developed in collaboration with an information specialist at the University of British Columbia, optimizing search terms, Boolean logic, and filters to maximize study retrieval. The screening process adhered to PRISMA guidelines, with independent review by two researchers, and a third researcher consulted to resolve discrepancies. Thus, our rigorous study selection methodology provides confidence that all eligible RCTs were comprehensively identified and included in our meta-analysis.

**Comment 8:** In the outcomes of interest, In the outcomes of interest, you indicate that a cardiac adverse event was defined as per the Medical Dictionary for Regulatory Activities terminology, but then do not give what this definition in.

**Response:** In our study, cardiac adverse events (CAEs) were defined using the Medical Dictionary for Regulatory Activities (MedDRA) terminology, ensuring a standardized classification of cardiovascular-related adverse drug reactions. According to MedDRA, CAEs encompass a range of clinically significant cardiovascular conditions, including cardiac arrhythmias (e.g., atrial fibrillation, bradycardia, ventricular tachycardia, QT prolongation), myocardial disorders (e.g., myocarditis, myocardial infarction), heart failure

syndromes (e.g., left ventricular dysfunction, cardiogenic shock), and conduction system abnormalities (e.g., heart block, bundle branch block).

To maintain consistency in classification, all reported CAEs in the included RCTs were systematically categorized using MedDRA codes, with a complete list provided in Supplementary Table 13. Furthermore, our classification methodology aligns with regulatory pharmacovigilance frameworks, such as the US FDA Adverse Event Reporting System (FAERS), which has been widely used to assess drug-induced cardiotoxicity. To enhance clarity, we have explicitly incorporated this definition into the manuscript.

**Comment 9:** What is the difference between your primary is the difference between your primary outcome of interest “cardiac adverse event” and your second outcome of interest “specific reported cardiac adverse event”?

**Response:** We appreciate the reviewer’s question. The primary outcome of interest in our study was a composite measure of CAEs, defined using the Medical Dictionary for Regulatory Activities (MedDRA) terminology. This composite outcome encompassed any cardiovascular event reported in the included RCTs, enabling assessment of the overall incidence of cardiac complications associated with remdesivir use. In contrast, the secondary outcome focused on specific reported cardiac adverse events, such as arrhythmias, heart failure, myocardial disorders, or mitral valvular disorders. Unlike the aggregate primary outcome, the secondary outcome allowed for a granular analysis of individual CAE subtypes, assessing potential differences in specific cardiovascular effects of remdesivir.

To ensure statistical robustness, the effect of remdesivir versus placebo and/or standard care on specific CAEs was analyzed only when these events were present in ≥5% of patients per treatment group, allowing for meaningful comparisons. To enhance clarity, we have revised the Outcomes of Interest section in the manuscript to explicitly differentiate between the composite primary outcome and the specific secondary outcomes.

**Comment 10:** Why were the effects of remdesivir vs placebo only looked at if the events occurred in more than 5% of patients per group? It could still be a meaningful analysis with a prevalence less than this.

**Response:** We appreciate the reviewer's comment and recognize the concern regarding the 5% prevalence threshold for analyzing specific cardiac adverse events (CAEs). This threshold was pre-specified to ensure statistical robustness and minimize the risks associated with rare event analysis, as outlined in Chapter 10 of the Cochrane Handbook for Systematic Reviews of Interventions (Dealing with rare events, Section 10.4). https://training.cochrane.org/handbook/current/chapter-10

Analyzing events with a very low prevalence (< 5%) presents several statistical challenges, including:

1) sparse data bias, which can distort effect estimates.
2) wide confidence intervals, reducing precision and increasing uncertainty.
3) unstable estimates in meta-analysis models, particularly with random-effects models.

This threshold was selected to optimize statistical reliability while maintaining clinical applicability, ensuring that event rates were sufficiently high to yield meaningful and interpretable effect estimates. This approach aligns with Cochrane Handbook recommendations, which caution that studies with very rare events may produce unreliable meta-analyses due to high heterogeneity and model instability. However, we acknowledge that lower-prevalence events may still have clinical significance. To address this, we have revised the Discussion section to include this as a potential limitation, noting that our approach may have excluded rarer but clinically relevant cardiac events.

**Comment 11:** For the subgroup analyses, can you list all baseline co-morbidities that were considered in the meta-regression.

**Response:** We appreciate the reviewer's comment. The baseline comorbidities considered in the meta-regression analyses included diabetes, cardiovascular disease, chronic lung disease, chronic liver disease, obesity, and chronic kidney disease, as reported in Supplementary Tables 10, 11, and 12. Due to low prevalence (<5%) of some comorbidities, we limited subgroup analyses to those with sufficient data across studies. Only studies reporting relevant subgroup data were included in each analysis. Meta-regression results are presented in Supplementary Figures 1, 2, and 3.

## VERSION 2 - REVIEW

| | |
|---|---|
| **Reviewer** | **5** |
| **Name** | **Martin, James** |
| **Affiliation** | **University of Birmingham, Institute of Applied Health Research** |
| **Date** | **19-Apr-2025** |
| **COI** | |

Thank you for this resubmission and for addressing the comments made by the reviewers.I have some minor comments that still remain:

In the abstract, you say that reviewers extracted data following the PRISMA-Harms guidelines. The PRISMA guidelines are for reporting and not guidelines on extracting data.

In the abstract, you have a sentence of “specific CAEs included arrhythmias, heart failure, and myocardial disorders.” This sentence needs more context, as whilst you have said elsewhere that these are secondary outcomes, that isn’t clear here. It also isn’t clear that the primary outcome is a composite, and includes any adverse cardiac event.

The abstract reports results about subgroup analysis, but no details about the statistical methodology are included for them.

You state that you report “weighted median and interquartile ranges”. You need to be clear about what weighting was used.

The primary analysis involves a random-effects regression model. For the sub-group analyses, you have used fixed-effects meta-regression. I wonder if you could elaborate as to why random-effects are used for the primary analysis, but fixed-effects used for the subgroup?

When looking at subgroup analysis, I was expecting either Covid 19 disease severity or Covid-19 vaccine uptake to be examined. Was this something that was looked at or is there rationale for not looking at it?

In the methods section, can you clearly report exactly what subgroups were looked at. It is not sufficient to say: “examining sex differences and baseline comorbidities such as cardiovascular disease and diabetes”. It is not clear from this whether you have looked at 3 subgroup analyses. You could easily have looked at 333 subgroups under this vague description.

## VERSION 2 - AUTHOR RESPONSE

**Reviewer # 5**

**1.** In the abstract, you say that reviewers extracted data following the PRISMA-Harms guidelines. The PRISMA guidelines are for reporting and not guidelines on extracting data.

**Response:** Thank you for your comments. We have revised the sentence in the Abstract. Please

see lines 46-47 of the revised manuscript.

**2.** In the abstract, you have a sentence of "specific CAEs included arrhythmias, heart failure, and myocardial disorders." This sentence needs more context, as whilst you have said elsewhere that these are secondary outcomes, that isn't clear here. It also isn't clear that the primary outcome is a composite, and includes any adverse cardiac event.

**Response:** Thank you for pointing this out. We have revised the Abstract to clearly distinguish between the composite primary outcome and the secondary outcomes. Please see lines 51-53 of the revised manuscript.

**3.** The abstract reports results about subgroup analysis, but no details about the statistical methodology are included for them.

**Response:** Thank you for your thoughtful input on the recommendations about subgroup analysis. We have already revised Methods section.

**4.** You state that you report "weighted median and interquartile ranges". You need to be clear about what weighting was used.

**Response:** Thank you for pointing this out. We calculated weighted medians and interquartile ranges using the number of participants per study as weights, to reflect the contribution of each study to the pooled patient population. This clarification has been added to the revised Methods section.

**5.** The primary analysis involves a random-effects regression model. For the sub-group analyses, you have used fixed-effects meta-regression. I wonder if you could elaborate as to why random-effects are used for the primary analysis, but fixed-effects used for the subgroup?

**Response:** We used a random-effects model for the primary analysis to account for between-study heterogeneity. For subgroup analyses, we applied fixed-effects meta-regression due to the small number of studies and limited power for random-effects modeling. These were exploratory analyses to examine whether study-level covariates could explain effect size variability. This rationale has been clarified in the revised Methods section.

**6.** When looking at subgroup analysis, I was expecting either Covid 19 disease severity or Covid-19 vaccine uptake to be examined. Was this something that was looked at or is there rationale for not looking at it?

**Response:** Thank you for this thoughtful question. Regarding vaccine uptake, all seven RCTs included in our meta-analysis were conducted before April 2021, and none of the participants

had received a COVID-19 vaccine. This allowed us to evaluate the cardiac safety of remdesivir independent of potential confounding from vaccine-associated myocarditis. While this limits generalizability to vaccinated populations, it aligns with our study objective to isolate remdesivir's cardiotoxic potential. Accordingly, we have added a forward-looking statement in the Discussion emphasizing the need for post-vaccination safety studies and real-world pharmacovigilance data to complement RCT findings (Lines 453-458).

As for COVID-19 disease severity, five of the seven trials reported baseline severity using WHO ordinal categories. Most patients were hospitalized with moderate illness (categories 3– 5), and notably, 63.4% of patients who experienced cardiac adverse events (CAEs) were classified as category 3-hospitalized but not requiring supplemental oxygen. This suggests that CAEs were not confined to the most critically ill patients. However, due to heterogeneous reporting formats and the lack of individual-level CAE data stratified by severity in the published RCTs, we were unable to conduct a formal subgroup meta-analysis by disease severity. We have acknowledged this as a methodological limitation in the revised manuscript and agree that this question warrants further investigation in future individual participant data (IPD) meta-analyses.

**7.** In the methods section, can you clearly report exactly what subgroups were looked at. It is not sufficient to say: "examining sex differences and baseline comorbidities such as cardiovascular disease and diabetes". It is not clear from this whether you have looked at 3 subgroup analyses. You could easily have looked at 333 subgroups under this vague description.

**Response:** Thank you for the suggestion. We have revised the Methods section to explicitly list the subgroups analyzed in the meta-regression: sex, cardiovascular disease, diabetes, chronic lung disease, chronic liver disease, and obesity. These variables were selected based on consistent reporting across the included studies.

We would like to thank the editors and reviewers once again for their valuable feedback. The manuscript and supplementary files have been revised accordingly. We appreciate your thoughtful comments, which have significantly improved the clarity and quality of our work. Thank you for your continued consideration.

---

## VERSION 3 - REVIEW

| | |
|---|---|
| **Reviewer** | **5** |
| **Name** | **Martin, James** |
| **Affiliation** | **University of Birmingham, Institute of Applied Health Research** |
| **Date** | **22-Jun-2025** |
| **COI** | |

Thank you for addressing the comments made.

I found that last sentence of the abstracts conclusion interesting: “Further research is warranted to validate these findings and explore potential mechanisms underlying remdesivir’s cardiotoxic effects.”. Given the RR is 0.84 (95%CI: 0.68 to 1.04), this would suggest a likely reduction in risk of CAE (which seems the opposite to this conclusion and the opposite to preliminary concerns). Whilst I am not suggesting you do this for this manuscript, you might (for a future publication) want to consider performing a Bayesian meta-analysis – particularly given the results you found seem to disagree with preliminary results.

## VERSION 3 - AUTHOR RESPONSE

2. Reviewer comments

Thank you for addressing the comments made. I found that last sentence of the abstracts conclusion interesting: “Further research is warranted to validate these findings and explore potential mechanisms underlying remdesivir’s cardiotoxic effects.”. Given the RR is 0.84 (95%CI: 0.68 to 1.04), this would suggest a likely reduction in risk of CAE (which seems the opposite to this conclusion and the opposite to preliminary concerns). Whilst I am not suggesting you do this for this manuscript, you might (for a future publication) want to consider performing a Bayesian meta-analysis – particularly given the results you found seem to disagree with preliminary results.

**Response:** Thank you for your continued review and for your thoughtful and helpful comments. We agree that the relative risk point estimate (<1.0) suggests a possible reduction in CAE risk, although the confidence interval includes the null and the result is not statistically significant.

In response, we have revised the abstract and discussion to reflect this nuance more accurately. A Bayesian meta-analysis may indeed offer valuable additional insights, particularly when

integrating prior information from observational studies and case reports. We appreciate this methodological suggestion and will consider it in future work.
